# Seasonal variability of lotic macroinvertebrate communities at the habitat scale demonstrates the value of discriminating fine sediment fractions in ecological assessments

**DOI:** 10.1002/ece3.10564

**Published:** 2023-09-29

**Authors:** Kate L. Mathers, Patrick D. Armitage, Matthew Hill, Morwenna McKenzie, Isabel Pardo, Paul J. Wood

**Affiliations:** ^1^ Geography and Environment Loughborough University Loughborough UK; ^2^ River Laboratory Freshwater Biological Association Wareham UK; ^3^ Department of Life and Environmental Sciences, Faculty of Science and Technology Bournemouth University Poole UK; ^4^ Department of Ecology and Animal Biology University of Vigo Vigo Spain

**Keywords:** beta diversity, biological traits, mesohabitat, nestedness, stability, taxonomic

## Abstract

Despite lotic systems demonstrating high levels of seasonal and spatial variability, most research and biomonitoring practices do not consider seasonality when interpreting results and are typically focused at the meso‐scale (combined pool/riffle samples) rather than considering habitat patch dynamics. We therefore sought to determine if the sampling season (spring, summer and autumn) influenced observed macroinvertebrate biodiversity, structure and function at the habitat unit scale (determined by substrate composition), and if this in turn influenced the assessment of fine sediment (sand and silt) pressures. We found that biodiversity supported at the habitat level was not seasonally consistent with the contribution of nestedness and turnover in structuring communities varying seasonally. Habitat differences in community composition were evident for taxonomic communities regardless of the season but were not seasonally consistent for functional communities, and, notably, season explained a greater amount of variance in functional community composition than the habitat unit. Macroinvertebrate biodiversity supported by silt habitats demonstrated strong seasonal differences and communities were functionally comparable to sand habitats in spring and to gravel habitats in autumn. Sand communities were impoverished compared to other habitats regardless of the season. Silt habitats demonstrated a strong increase in Ephemeroptera, Plecoptera and Trichoptera (EPT) taxa and functional richness from spring into autumn, while vegetation habitats displayed a peak in EPT abundance in summer. Only silt and sand habitats demonstrated temporal variability in functional evenness suggesting that these habitats are different in terms of their resource partitioning and productivity over time compared to other habitats. Gravel and vegetation habitats appeared to be more stable over time with functional richness and evenness remaining consistent. To accurately evaluate the influence of fine sediment on lotic ecosystems, it is imperative that routine biomonitoring and scientific research discriminate between sand and silt fractions, given they support different biodiversity, particularly during summer and autumn months.

## Graphical abstract

We sought to examine if seasonal sampling (spring, summer or autumn) influenced observed macroinvertebrate biodiversity, structure and function at the habitat unit scale (as determined by substrate composition) and if this in turn influenced the assessment of fine sediment (sand and silt) pressures on instream ecology. Biodiversity supported at the habitat level was not seasonally consistent when considered within the wider habitat provision at the reach scale particularly for functional (biological traits) level. In particular, results suggest that silt habitats are perhaps not as impoverished as previously perceived when considered seasonally.

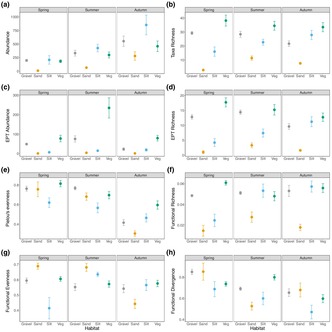

## 1 INTRODUCTION

Lotic systems vary spatially and temporally associated with flow regime variability (Lytle & Poff, 2004; Sofi et al., 2020), instream primary productivity (Cotton et al., 2006; Lürig et al., 2021) and sediment inputs (Davis et al., 2022; Sherriff et al., 2018). Furthermore, riverine macroinvertebrate populations and communities display strong seasonal variability linked with their voltinism which results in fluctuations in community assemblages over annual and multi‐annual timescales (Beche et al., 2006; Hynes, 1972; Mazor et al., 2009). This seasonal turnover in macroinvertebrate populations may be more influential in explaining community variability than sampling site characteristics (Jensen et al., 2021). However, despite the widely recognised temporal dynamism of both abiotic and biotic components of riverine systems, few studies have considered the potential influence of season on their study outcomes, with most sampling being conducted on a single occasion, during a designated time period or being assessed as annual mean values (but see Helms et al., 2009). The timing of ecological appraisal may have significant implications for the interpretation of observed results and for management recommendations for fine sediment pressures, the flow regime, or wider river restoration activities (Carlson et al., 2013; Johnson et al., 2012; Linke et al., 1999). Indeed, Ormerod (1987) indicated that the most comprehensive approach to characterise riverine biodiversity was by employing a combined habitat scale (pool, riffle and marginal) and seasonal sampling strategy.

Fine sediment (<2 mm) has been widely acknowledged to act as a master environmental filter in shaping lotic macroinvertebrate communities at the landscape (Davis et al., 2022; dos Reis Oliveira et al., 2018) and local/patch scale (Descloux et al., 2013; Mathers et al., 2017). Although fine sediments are a natural component of lotic ecosystems, contemporary fine sediment loading far exceeds historic levels. It is anticipated that fine sediment inputs will be further exacerbated in the future due to changes in climatic driven runoff regimes and intensification of agricultural practices in response to globally increasing food production demands (Burt et al., 2016; Collins & Zhang, 2016). Therefore, understanding the role that fine sediment deposits play in supporting taxonomic and functional biodiversity will improve the knowledge base to support effective environmental monitoring strategies in the future. Currently the majority of riverine biomonitoring and assessment tools are developed and undertaken at the meso‐scale (multiple riffle/pools; e.g. Salmaso et al., 2021), however it is likely that fine sediment dynamics will exhibit stronger associations with instream communities at the habitat scale (Larsen et al., 2009).

Rivers can be delineated via various hierarchal spatial scales and criteria ranging from reach and riffle/pools to habitat (substrate) and micro‐habitat (individual particle) patches (Armitage, 2006). The habitat unit represents a spatial scale particularly relevant for management as it can be rapidly identified through visual bankside assessments of discrete patches of substrate (e.g. vegetation, gravel, sand, silt; Armitage & Cannan, 2000). Substrate composition is widely acknowledged to be a key driver in controlling macroinvertebrate community structure (Beisel et al., 2000; Richards et al., 1993), though much less has been documented regarding the influence on functional communities (but see Demars et al., 2012; White et al., 2017), and there is an absence of information on seasonal trajectories of functional communities.

Biological functional traits are based on the habitat model concept (after Southwood, 1977), and therefore it is highly likely that community traits may reflect spatial and temporal variations in the physical environment much more strongly than taxonomic identity (Schmera et al., 2013). Given their theoretical foundation within the habitat template, it is surprising that there remains little evidence on the association of functional communities with habitat units and how these vary temporally. Trait‐based approaches enable mechanistic understanding of the processes taking place in lotic ecosystems and thereby facilitate recognition of how environmental change may affect natural functioning. In recent years, functional measures have been applied to a range of environmental stressors including flow regime modifications (Belmar et al., 2019; White et al., 2021), invasive species (Guareschi et al., 2021; Mathers et al., 2020), excessive fine sedimentation (Juvigny‐Khenafou et al., 2021; Mathers et al., 2022) and the evaluation of instream restoration measures (England & Wilkes, 2018; Magliozzi et al., 2020). However, habitat structure and availability has been shown to moderate the ecological effects of stressors and that the communities inhabiting distinct habitat units may react differently to instream measures such as river restoration practices (Brunke et al., 2001; Calapez et al., 2021; White et al., 2017). As such enhancing our evidence base of the functional communities inhabiting differing habitat units temporally will facilitate identification of the potential effects of anthropogenic and natural stressors for ecosystem functioning.

To address the knowledge deficit regarding the seasonal variability in macroinvertebrate communities at the habitat scale, this study aims to characterise the biodiversity supported within four habitat units (characterised by substrate composition: vegetation, gravel, sand and silt) over the course of three seasons (spring, summer and autumn). We examined taxonomic and functional facets of macroinvertebrate alpha and beta diversity. Establishing this knowledge base is vital to enable scientists and managers to accurately appraise ecological health across the entire year and ensure management recommendations are effective across seasons. In particular, we tested the following hypotheses:

1. There will be seasonal differences in observed taxonomic and functional macroinvertebrate alpha and beta diversity associated with habitat units.
2. Fine sediment sand and silt habitat units will differ in the macroinvertebrate diversity they support and should be considered independently within ecological assessments.
3. Macroinvertebrate communities inhabiting fine sediment habitat units (sand vs. silt) will display the strongest seasonal variability of all habitat units considered.

## 2 MATERIALS AND METHODS

### 2.1 Macroinvertebrate data

One set of macroinvertebrate samples were collected from a 300‐m reach of the Mill stream, located in Dorset, UK over three seasons in 1992: spring (late April), summer (early July) and autumn (late September). Winter was not sampled due to high‐water levels typically encountered during these months. Four habitat units were visually identified in each sampling season and each represented discrete patches of substrate/vegetation as opposed to a gradient of substrate sizes or vegetation species. The four habitats examined in each season were: (a) gravel, (b) sand, (c) silt and (d) perennial vegetation (marginal perennial emergent *Phragmites* sp.). Other individual vegetation habitats (e.g. *Nasturtium*, *Ranunculus*) were present in the reach but have not been included herein. On each occasion 10 replicate samples were taken for each substrate habitat; only five silt samples were taken during spring due to its naturally low occurrence during this season, resulting in a total of 115 samples. Samples consisted of a 15‐s kick/sweep sample using a 900‐μm pond net following the method outlined in Armitage et al. (1995), focusing on the habitat patch visually identified and delineated prior to sampling. All samples were preserved in 5% formalin in the field and identified to species level in the laboratory wherever possible. Diptera (including Chironomidae) were mostly recorded to genus level resulting in a total of 184 taxa (37,574 individuals). Data utilised in this study comprise part of that presented in Pardo and Armitage (1997) and Armitage et al. (1995; note only the category termed gravel ‘fast’ was utilised as gravel in this study, and all other vegetation categories were not analysed: *Nasturtium*, *Ranunculus* fast, *Ranunculus* slow). Although the data herein represent a re‐analysis of a previously published dataset, analysis and findings presented differ substantially in their focus (fine sediment dynamics) in addition to contemporary biodiversity analyses conducted (beta diversity, biodiversity alpha metrics and entire functional component) being new.

### 2.2 Functional traits

Macroinvertebrates were assigned to 11 biological ‘grouping features’ comprising 63 functional traits from the Tachet et al. (2010) European trait database (Table S1). The database employs a fuzzy coded procedure with faunal affinities to individual traits ranging from zero (*no affinity*) to three or five (*high affinity*). Trait values were therefore standardised following ‘fuzzy coding’ standardisation (Chevene et al., 1994) using the *prep.fuzzy* function in the ade4 package (Thioulouse et al., 2018) so that each grouping feature summed to 1 (to ensure trait affinities had an equal weighting between taxa). Taxa recorded at a lower resolution than that of the trait database (e.g. species level) were aggregated and for taxa recorded to a higher level than the database (e.g. family level) affinities of all genera recorded were averaged to provide a family score (sensu Gayraud et al., 2003). A total of 82 (of 184) taxa were not assigned indervidual functional traits, the majority of which were Diptera, in particular Chironomidae and Simuliidae, which were recorded to a finer resolution than that in the trait database and therefore were aggregated to the higher level. Subsequently a Taxa × Traits compositional matrix was constructed using community weighted means via the *functcomp* function in the FD package (Laliberté et al., 2014) and used in subsequent analyses.

### 2.3 Statistical analyses

#### 2.3.1 Seasonal differences in taxonomic and functional composition associated with habitat classification

Seasonal taxonomic and functional compositional differences associated with the four substrate habitats were visually examined via non‐metric multidimensional scaling (NMDS) using the *metaMDS* function in the vegan package (Oksanen et al., 2019). Statistical differences in community composition (taxonomic and functional) associated with habitat, season and their interaction were tested via permutational multivariate analysis of variance (PERMANOVA) using the *adonis* function in the vegan package. Where significant differences occurred by habitat, pairwise comparisons of differences were performed using the *pairwise.adonis* function for each individual season (Arbizu, 2019). Indicator species for each habitat classification were identified using the *multipatt* function within the indicspecies package (De Cáceres et al., 2020). An indicator value of >0.25 was accepted as ecologically relevant (Dufrêne & Legendre, 1997), and all significant indicators with a fidelity value of <0.25 were removed to exclude rare taxa (De Cáceres et al., 2012).

To test for potential seasonal differences in the heterogeneity of macroinvertebrate community composition (beta diversity) of each habitat classification, homogeneity of multivariate dispersions were calculated for functional and taxonomic communities using the *betadisper* function in vegan. Statistical differences in multivariate dispersion between seasons for each habitat were tested using one‐way ANOVA. Where significant seasonal differences occurred for a habitat, pairwise comparisons of differences were tested via Tukey's post hoc tests. Total beta diversity was decomposed into its nestedness and turnover components to investigate the dominant processes structuring macroinvertebrate taxonomic and functional composition. Prior to functional beta analysis the Taxa × Traits matrix underwent hierarchical clustering using the unweighted pair group method with arithmetic mean (UMPGA in the phangorn package; Schliep, 2011) using Gower distances following Cardoso et al. (2015). Mean taxonomic and functional total beta diversity were calculated and partitioned into mean turnover and nestedness components within each individual habitat (e.g. sand, silt) using the *beta.multi* function in the BAT package (Cardoso et al., 2021). Taxonomic and functional total beta diversity, nestedness and turnover pairwise distance matrices were calculated using the function *beta* in the BAT package. Subsequently, the mean contribution of nestedness and turnover for each habitat pairwise comparison (e.g. sand vs. silt) were calculated.

#### 2.3.2 Seasonal differences in taxonomic and functional alpha diversity associated with habitat classification

Five taxonomic metrics were derived: community abundance, taxa richness, richness and abundance of Ephemeroptera, Plecoptera and Trichoptera (EPT) taxa and Pielou's evenness. Three functional metrics were calculated comprising functional richness (FRic) representing the minimum convex hull encompassing all species, functional evenness (FEve) reflecting the regularity in which species are distributed across functional space and functional divergence (FDiv) representing how abundance is distributed within the volume of functional space occupied by species (Villéger et al., 2008). The three functional diversity metrics were computed using the *dbFD* function on a Gower dissimilarity matrix in the FD package. General linear models (GLM) were constructed for each response variable by dataset and were fitted with the fixed interacting effects of habitat and season using the *glm* function in the stats package. All models were fitted with a Gaussian error distribution with the exception of abundance, taxa richness, EPT richness and EPT abundance which were fitted with a Poisson error distribution. Significant differences associated with habitat and season were tested via post hoc pairwise comparisons of groups using estimated marginal means, and *p* values were adjusted for multiple comparisons via Tukey's tests within the emmeans package (Lenth et al., 2020). All analyses were conducted in the R Environment (R Development Core Team, 2022).

## 3 RESULTS

### 3.1 Seasonal differences in taxonomic and functional composition associated with habitat classification

**FIGURE 1:**
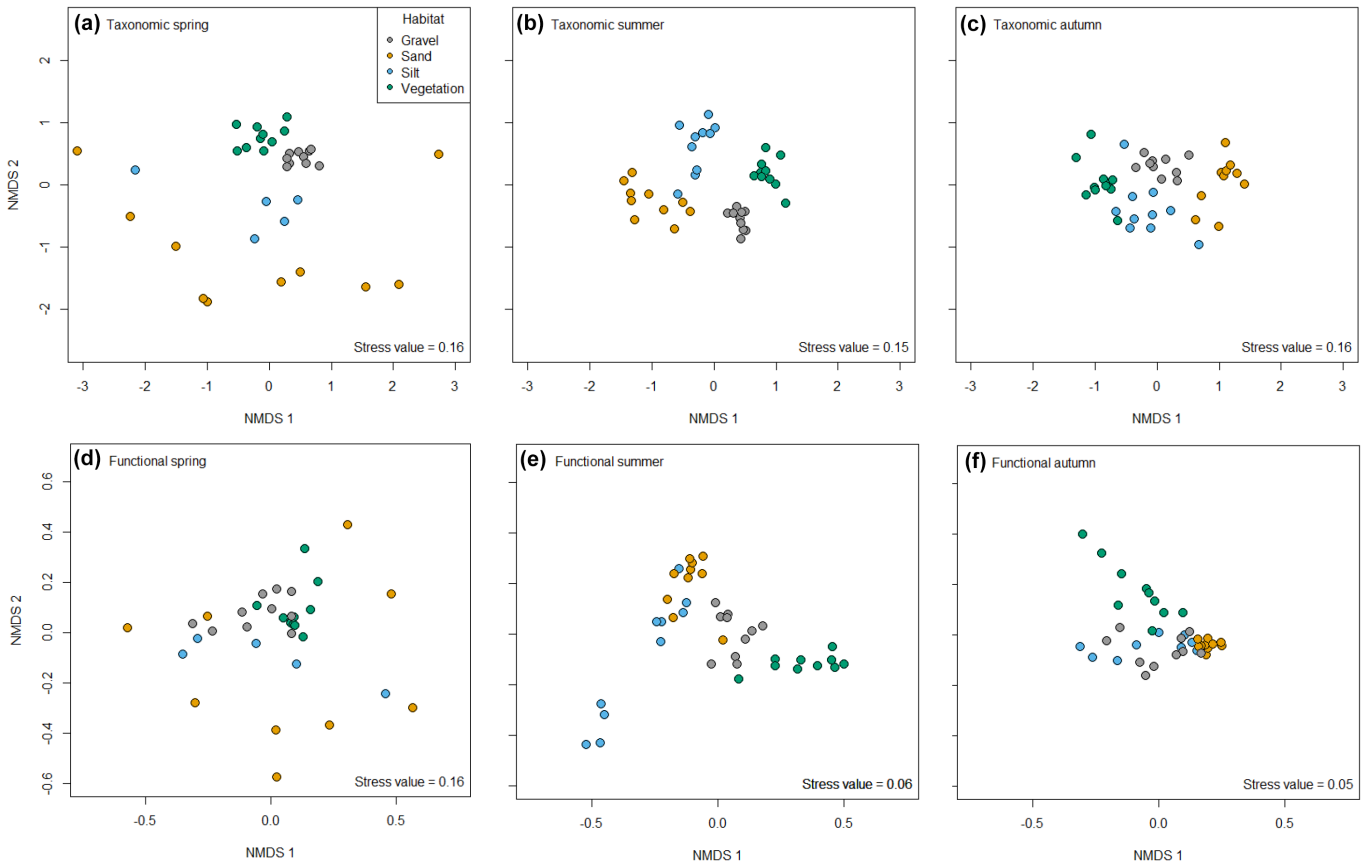
Non‐metric multidimensional scaling (NMDS) of macroinvertebrate community composition associated with four habitat types (gravel, sand, silt and vegetation) in the Mill Stream, UK in spring, summer and autumn for (a–c) taxonomic and (d–f) functional communities.

PERMANOVA analyses indicated that habitat explained the greatest amount of variation (associated with the highest *r* ^2^ value) in taxonomic composition, while season explained the most variation for functional composition (Table 1). In the case of taxonomic communities, habitat and season exerted a similar influence on community composition, while for functional communities' season was more influential (as denoted by the highest *F* values). In both instances the interaction of habitat and season were statistically significant (Table 1). Habitat differences in taxonomic and functional community composition were strongest in summer with all pairwise comparisons being significant (Figure 1; Table 2). Taxonomic communities demonstrated significant differences in community composition for all pairwise habitat comparisons in all seasons (Figure 1; Table 2). In contrast, there were no significant differences in functional communities inhabiting sand and silt habitats in spring, or silt and gravel habitats in autumn, with all other pairwise habitat comparisons being statistically significant (Figure 1; Table 2).

**TABLE 1:** Summary of PERMANOVA output assessing the relative importance of the habitat, season (spring, summer, autumn) and their interaction on taxonomic and functional macroinvertebrate communities.

	Taxonomic			Functional		
	*F*	*r* ^2^	*p*	*F*	*r* ^2^	*p*
Habitat	14.65	20.61	**<.001**	15.29	19.52	**<.001**
Season	18.61	17.45	**<.001**	29.11	24.76	**<.001**
Habitat × Season	4.85	13.65	**<.001**	4.67	11.91	**<.001**

*Note:* Significant (*p* < .05) results are in bold.

**TABLE 2:** Summary of pairwise PERMANOVA testing for statistical differences between habitat types.

Factor	Spring			Summer			Autumn		
	*F*	*r* ^2^	*p*	*F*	*r* ^2^	*p*	*F*	*r* ^2^	*p*
Taxonomic									
Silt versus vegetation	4.40	25.30	**.002**	15.51	46.29	**.001**	4.17	18.81	**.002**
Silt versus sand	2.63	16.83	**.002**	12.08	40.15	**.001**	6.76	27.29	**.002**
Silt versus gravel	4.87	27.24	**.001**	9.97	35.66	**.001**	2.45	12.00	**.032**
Vegetation versus sand	5.84	24.49	**.001**	24.45	57.60	**.001**	6.77	27.33	**.001**
Vegetation versus gravel	6.23	25.70	**.001**	13.38	42.64	**.001**	5.17	22.31	**.003**
Sand versus gravel	7.66	29.85	**.001**	15.92	46.94	**.001**	5.04	21.88	**.021**
Functional									
Silt versus vegetation	5.51	29.77	**.001**	15.62	46.46	**.001**	5.12	22.14	**.001**
Silt versus sand	1.24	8.70	.285	7.43	29.21	**.001**	8.42	31.86	**.001**
Silt versus gravel	2.94	18.43	**.022**	10.40	36.62	**.001**	1.43	7.36	.203
Vegetation versus sand	6.18	25.55	**.001**	21.92	54.91	**.001**	18.15	50.22	**.001**
Vegetation versus gravel	5.95	24.83	**.001**	13.18	42.28	**.001**	10.58	37.02	**.001**
Sand versus gravel	4.57	20.25	**.003**	12.42	40.84	**.001**	12.94	41.81	**.001**

*Note*: Significant (*p* < .05) results are in bold.

Indicator species analysis indicated that vegetation habitats supported the greatest number of indicator species (18–20) regardless of the season with these species being a mixture of taxonomic orders (Table S2). Gravel habitats supported the second greatest number of indicator species in spring (17) and autumn (19), but the number of species identified in autumn dropped considerably to eight. Only Diptera taxa were identified for silt habitats in spring (8 taxa) which broadened in summer to support several orders (13 indicators; Table S2). In autumn, silt habitats supported the second highest number of indicators, and this comprised a range of orders including three trichopteran species (*Mystacides azurea*, *Molanna angustata* and *Athripsodes cinereus*). Sand habitats did not support any indicator species in spring, but two chironomid genera were identified in summer (*Chironomus* spp., *Stictochironomus* spp.) and one genus in autumn (*Stictochironomus* spp.; Table S2).

Overall, taxonomic and functional beta diversity of the entire reach was most heterogeneous in spring with autumn being the most homogeneous (Table 3; Table S3; Figure 1). Statistical differences in taxonomic community heterogeneity between habitats was only evident in spring (Table S4) with sand communities being significantly more variable than gravel (*p* < .001) and vegetation (*p* = .001) habitats (Table 3). In contrast, functional communities were heterogeneous between habitats in all three seasons (Table S4). In spring, sand communities demonstrated greater functional heterogeneity than gravel and vegetation (both *p* < .001; Table 3). In summer and autumn, statistical differences between sand and silt habitats (summer *p* = .031 and autumn *p* = .010) were evident with silt supporting greater functional heterogeneity (Table 3). Sand habitats demonstrated temporal variation in beta diversity for both taxonomic and functional macroinvertebrate communities, while gravel habitats demonstrated temporal variability for functional communities (Table 4). Pairwise comparisons of sand taxonomic and functional communities and gravel functional communities indicated that heterogeneity was greatest in spring and was reduced in summer and autumn (Table S5; Table 3).

**TABLE 3:** Summary of mean multivariate dispersion distance by habitat for taxonomic and functional macroinvertebrate communities in spring, summer and autumn.

	Spring	Summer	Autumn
Taxonomic			
Silt	0.466	0.394	0.379
Sand	0.611	0.307	0.273
Gravel	0.343	0.335	0.293
Vegetation	0.409	0.350	0.376
Functional			
Silt	0.129	0.119	0.094
Sand	0.196	0.064	0.026
Gravel	0.084	0.072	0.056
Vegetation	0.078	0.083	0.073

**TABLE 4:** Summary of ANOVA permutation dispersion tests between seasons for individual habitat classifications for taxonomic and functional macroinvertebrate communities in UK chalk streams.

Habitat	*F*	*p*
Taxonomic		
Silt	0.58	.571
Sand	24.79	**<.001**
Gravel	0.66	.525
Vegetation	0.43	.653
Functional		
Silt	0.89	.426
Sand	41.93	**<.001**
Gravel	3.59	**.042**
Vegetation	0.19	.831

*Note*: Significant (*p* < .05) results are in bold.

**FIGURE 2:**
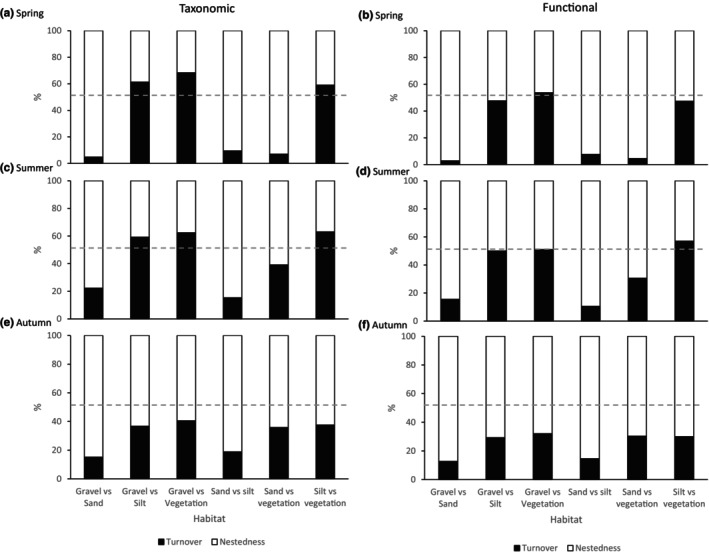
Mean contribution of nestedness and turnover to the total beta diversity for pairwise comparisons between the four habitat types for spring, summer and autumn for taxonomic (a, c, e) and functional (b, d, f) macroinvertebrate communities. The grey dashed line indicates when both processes contribute equally to beta diversity.

When beta diversity was decomposed into its nestedness and turnover components, pairwise comparisons between sand and other habitats demonstrated a strong contribution of nestedness for both taxonomic and functional communities in all seasons (Figure 2). Nestedness was more prominent in spring for sand communities with the contribution of turnover increasing in summer and autumn (Figure 2). Autumn communities displayed greater contributions of nestedness regardless of the biodiversity facet or habitat considered (Figure 2). Sand communities demonstrated the highest levels of nestedness when beta diversity was decomposed within each habitat type (80% for taxonomic and 84% for functional) followed by silt (64% and 70% respectively), gravel (52% and 62% respectively) and vegetation (50% and 37% respectively; Table S6).

### 3.2 Seasonal differences in taxonomic and functional alpha diversity associated with habitat classification

**FIGURE 3:**
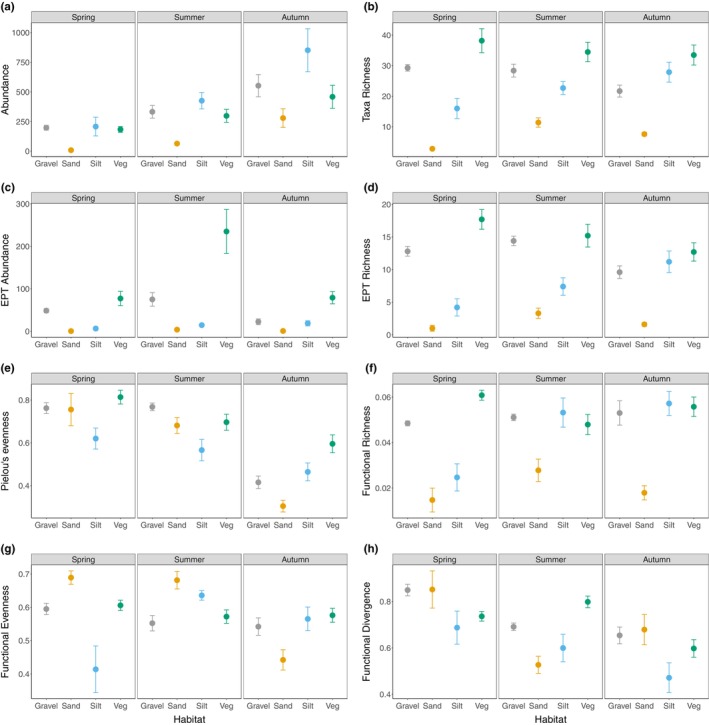
Mean (±1 SE) (a) abundance, (b) taxa richness, (c) EPT abundance, (d) EPT richness, (e) Pielou's evenness, (f) functional richness, (g) functional evenness and (h) functional divergence of macroinvertebrate communities in the four studied habitat types (gravel, sand, silt and vegetation) in spring, summer and autumn.

All eight biodiversity community metrics demonstrated a significant Habitat × Season interaction (*p* < .05). Only sand habitats displayed seasonal variations in community abundance with statistically lower values in spring compared to all other habitats (Tables S7 and S8). There were no significant differences in abundances between the habitats for summer or autumn (Table S7), though all four habitats demonstrated greatest abundance in autumn with the lowest values in spring (Figure 3). Taxa richness exhibited seasonal variations for sand (all seasons were different), gravel (reduced values in autumn) and silt (reduced values in spring), while vegetation demonstrated no significant differences among seasons (Table S7; Figure 3). EPT abundance displayed a notable peak in vegetation during the summer (Figure 3). Silt displayed a seasonal increase in EPT richness, while gravel and vegetation demonstrated reduced values in autumn compared to spring and summer (Figure 3; Table S8). All pairwise comparisons of EPT richness were significant in spring, but silt exhibited comparable richness as gravel and vegetation in autumn (Table S7). In general, Pielou's evenness demonstrated reductions in values in autumn compared to spring with this being notable for sand habitats in particular (Figure 3). Only silt habitats demonstrated seasonal variation in functional richness with values increasing significantly from spring to autumn resulting in no significant differences compared to gravel and vegetation habitats in summer and autumn (Figure 3; Tables S7 and S8). Both fine sediment habitat types (silt and sand) displayed temporal changes in functional evenness over time (Table S8). Functional evenness increased in summer and autumn relative to spring in silt habitats and was reduced in autumn in sand habitats (Figure 3). Silt habitats supported significantly lower functional evenness relative to other habitats (Table S7) in spring, while sand habitats supported lower evenness than gravel and vegetation in summer and all habitats in autumn (Table S7). All habitats demonstrated seasonal variations in functional divergence values (Table S8) with pairwise differences between habitats being evident during summer and autumn but not spring (Figure 3; Table S7). Overall silt habitats demonstrated the lowest functional divergence value which was recorded in autumn (Table S7).

## 4 DISCUSSION

Biotic and abiotic components of riverine systems display high levels of seasonal variability (Power et al., 1988). Despite this, much of the scientific research continues to be based on spot sampling, and biomonitoring approaches typically do not consider seasonal variability when assessing riverine condition (with site metrics often being a mean annual value). Our results indicate that season is a significant factor influencing macroinvertebrate biodiversity at the substrate habitat unit which should be acknowledged when interpreting survey results, providing support for our first hypothesis. Both taxonomic and functional community composition demonstrated a significant interaction of habitat with season, indicating that the structure and function of macroinvertebrate communities within habitat units are highly dependent on the season samples that were collected. In particular, summer represented the season where differences between habitats were most clear for both taxonomic (following Armitage et al., 1995) and functional communities. However, in the case of functional community composition, the influence and proportion of variance explained by season was greater than that of the habitat unit, while taxonomically the influence of season and habitat were comparable. Habitat differences in community composition were evident regardless of the season for taxonomic communities, supporting historic evidence of the importance of considering the spatial scale of the habitat unit when characterising biodiversity patterns (Armitage et al., 1995; Harper & Everard, 1998; Kemp et al., 2000; Newson & Newson, 2000). In marked contrast, discrete functional communities associated with the habitat unit were not evident for all seasons. Silt communities were functionally comparable to sand habitats in spring and to gravel habitats in autumn. As such, the assumption that habitat units and the environmental conditions that characterise them (e.g. substrate composition and flow velocity) support functionally discrete communities regardless of the sampling season may be unfounded. Beche et al. (2006) similarly reported that trait communities were functionally variable due to changing flow regime conditions and associated habitat availability.

Silt habitats were found to support highly unique taxonomic and functional assemblages, likely being strongly associated with flow velocities and as such may fluctuate in terms of their physical composition and longevity more readily than other habitat types including sand. Therefore, the communities present within fine sediment habitats are likely to be seasonally transient; something which is typically not considered when evaluating riverine health associated with fine sediments, particularly when other instream habitats are available immediately adjacent. Functionally, silt and sand habitats shared similar macroinvertebrate composition in spring but not during the other seasons, providing support for our second and third hypotheses that fine sediment habitats differ in the biodiversity they support and that this may vary seasonally. River discharge in temperate rivers demonstrates distinct seasonal patterns with the potential occurrence of multiple rising and falling limbs in spring (Worrall et al., 2014). As such this may lead to frequent deposition and erosion of fine sediments (Heywood & Walling, 2003), and therefore rapid turnover of habitat units during this season. Indeed, we observed comparable functional richness in silt and sand habitats in spring but not in other seasons. Moreover, indicator analysis identified only Diptera taxa as indicators for silt habitats with no indicator species identified for sand habitats, providing further evidence regarding the relatively harsh conditions that these fine sediment depositional habitats represent in spring.

Silt habitats were the only habitat to display temporal variations in functional richness, gaining richness over summer and into autumn, with functional richness values being comparable to gravel and vegetation habitats in both the summer and autumn (H3). The flow regime in temperate zone rivers during autumn months are typically stable following low and base flow conditions (Worrall et al., 2014), and it is highly probable that these conditions enabled community composition to be functionally comparable in gravel and silt habitats as we observed. Beta diversity values support this interpretation with community heterogeneity of the entire reach being highest for both functional and taxonomic communities in spring when flow regimes are more variable in temperate rivers and reach community heterogeneity being reduced in autumn months. In line with functional richness, we observed a seasonal increase in EPT and taxa richness in silt habitats over the summer with the highest values observed in autumn (driven by low representation of multiple EPT taxa naturally associated with their lifecycles). In vegetation habitats, the majority of community and functional biodiversity metrics remained high throughout the three sampling seasons. In addition, the number and diversity of indicator species identified for silt habitats increased in the summer and autumn from only 8 dipteran species being identified in spring to 13 and 14 indicator species during summer and autumn which encompassed a range of other orders and groups.

The comparable functional richness and composition of silt and gravel habitats in autumn may reflect increased occupancy of the ‘less ecologically optimal’ silt habitats during autumn as biotic competition in vegetation and gravel habitats remained high. In contrast to the common assumption that silt habitats are functionally impoverished (Buendia et al., 2013; Descloux et al., 2014), our results indicate that they can be compositionally unique habitats that are temporally dynamic with seasonally distinct community function and biodiversity. Under some environmental conditions, such as low/base flows when traditional biomonitoring often takes place in many temperate regions, silt habitats support functionally comparable communities to other habitat units, such as gravel and vegetation, that are often perceived more favourable for aquatic macroinvertebrates based on their taxonomic composition. The seasonally changing functional composition of silt communities may also provide some explanation as to why trait–sediment relationships are not straightforward to interpret (Wilkes et al., 2017), with season exerting more of an influence on functional community structure than environmental/habitat (substrate) conditions in this study.

Excessive fine sediment deposition has been widely reported to reduce EPT richness and abundance, with many of these taxa being characterised as being highly sensitive to fine sediment (Angradi, 1999; Descloux et al., 2013; Matthaei et al., 2006). Indeed, many studies examining the implications of fine sediment employ this metric as an indicator of lotic ecosystem health (Kaller & Hartman, 2004; Larsen et al., 2009). Although there were differences in EPT richness and EPT abundance in silt habitats when compared to all habitats in spring and summer, we found that sampling in autumn resulted in no significant differences for either metric when compared to gravel or vegetation habitats. In addition to biotic competition/resource depletion over the summer months leading to the potential migration of some taxa into the less optimal silt habitats, taxon life histories may also explain the absence of significant differences between gravel and silt habitats in autumn months with some taxa such as EPT being present as earlier life stages (eggs). Mathers et al. (2017) recorded differences in the strength of the fine sediment effect on sediment sensitive taxa (as defined by Turley et al., 2016) over the summer months, with this being linked to temporal lifecycle features of EPT taxa. In our study, indicator analysis indicated weaker preferences for gravel habitats in autumn months with the number of indicator species being reduced from 19 in the summer to only 8 in autumn. For example, two stonefly species (*Leuctra fusca* and *Leuctra geniculata*) which were recorded in high abundances in the summer in gravel habitats, and as such were identified as indicators, were absent in gravel in the autumn with only a few individuals being recorded in the vegetation. As such, studies and routine biomonitoring investigating the ecological implications of fine sediment should consider the potential influence of season in their findings and metric derivation, as we observed differing outcomes when different seasons were considered. Indeed, three species of trichopteran were determined as indicator species in silt habitats in autumn, a surprising finding given that one species, *Athripsodes cinereus*, was classified as moderately sensitive to fine sediment in one biomonitoring tool (Turley et al., 2016).

Sand habitats supported impoverished macroinvertebrate communities regardless of the season with significantly reduced abundance, taxa richness, EPT abundance, EPT richness and functional richness when compared to all habitats and seasons (except functional richness of silt habitats in spring). However, it should be noted that application of finer resolution taxonomic data does enable identification of potential indicator species that would otherwise be overlooked, given the majority of taxa inhabiting sand habitats are generalists that can tolerate a range of conditions. Two genera of chironomid (*Chironomus* and *Stictochironomus*) were identified as potential indicator species with the latter being identified in both summer and autumn. Typically, studies examining fine sediment deposition (<2 mm) do not discriminate between sand and silt fractions (but see Blöcher et al., 2020; Demars et al., 2012). However, our results clearly indicate that sand habitats are taxonomically and functionally different to silt habitats and as such the biodiversity is not comparable (supporting H2). The mechanisms driving these differences are likely linked to the particle size (Mathers et al., 2019) and the proportions of organic and inorganic particles (McKenzie et al., 2022). To accurately evaluate the potential implications of fine sediment on lotic ecosystem health, it is imperative that routine biomonitoring and scientific research discriminate between sand and silt fractions. This could be readily achieved even at the visual level (as demonstrated here), with many biomonitoring programmes already visually discriminating between sand, silt and clay fractions but with the data subsequently amalgamated to <2 mm. By retaining a finer sediment resolution, not only would this enable more accurate identification of the biodiversity supported at the habitat unit level and enhance the effectiveness of conservation and management efforts, it would also facilitate further research into the mechanisms driving the structure and function of macroinvertebrates inhabiting these two different fine sediment habitat units.

Vegetation represented the most temporally stable habitat in terms of its contribution to biodiversity with relatively high abundance and richness of taxa being supported during all seasons. It should be noted that this is most likely a function of the type of vegetation examined, with phragmites being a perennial species. Other vegetation species may support different seasonal trajectories (see Armitage et al., 1995; Pardo & Armitage, 1997). Only EPT abundance displayed a strong peak in summer months (driven strongly by *Brachycentrus subnubilus* that are a typical species of vegetation stands in this system; Gunn, 1985), with the majority of the other community and functional metrics values remaining high throughout the three sampling seasons. Vegetation stands are widely considered to represent heterogeneous habitats associated with increased structural complexity and interactions with the flow regime (Ferreiro et al., 2013; Wolters et al., 2018). In this study, vegetation habitat units demonstrated no significant variations in beta diversity with community composition remaining stable regardless of the biodiversity facet considered. However, when beta diversity was decomposed, functional vegetation communities were structured predominately by turnover (63%), in marked contrast to all other habitats that exhibited strong nestedness (63% for gravel, 70% for silt and 84% for sand habitats). The strong contribution of turnover in functional community composition supports the suggestion that vegetation is structurally complex, and the micro‐conditions (flow velocity and trapping of fine sediments) thereby support heterogeneous functions, which also likely explains its high functional richness across all seasons.

Seasonal differences in the processes structuring beta diversity between habitats were evident, with the contribution of nestedness and turnover in structuring communities varying seasonally (H1). The limited number of studies which have examined the processes structuring beta diversity in fine sediment habitats have reported that the communities were shaped by nestedness (Doretto et al., 2017, 2021; Mathers et al., 2022; Salmaso et al., 2021), which was also partly supported here. The processes shaping beta diversity therefore likely reflect the differences in richness among the different habitat units. Sand habitats remained the most taxonomically and functionally impoverished regardless of the season. In marked contrast, silt habitats demonstrated seasonal differences in functional and taxonomic richness with the greatest values being supported in autumn. However, our results suggest that the contribution of nestedness and turnover is not seasonally consistent, reflecting variation in abiotic, biotic and spatial conditions of the individual habitat units.

We observed strong temporal variations in functional divergence for silt and sand habitats. Functional divergence has been identified as an indicator of environmental stress associated with land use (Barnum et al., 2017; Martins et al., 2021). Traits most sensitive to land use disturbance will typically lie on the fringes of trait space and thus are most likely to be the first to be lost, resulting in a reduction in functional divergence values (Mouillot et al., 2013). Low functional divergence values are indicative of resource efficiency being low (Mason et al., 2005), while low functional evenness, as observed in silt habitats in spring, suggests that some parts of the trait niche space, though occupied are underutilised. We observed increased functional evenness in silt habitats from spring into the autumn in tandem with reductions in functional divergence. This suggests that communities were more readily utilising the functional niche provided by silt habitats and enhancing their productivity (supporting the theory of taxa migrating to silt habitats from other habitat units during autumn), but that resources were limited, and competition was high. Only silt and sand habitats demonstrated temporal variability in functional evenness suggesting that these habitats are different in terms of their resource partitioning and productivity over time compared to other habitats (H3).

Our study has demonstrated that the habitat unit is a significant parameter in structuring biodiversity. By conducting research at this spatial scale, we have been able to elucidate on the mechanisms controlling reach scale biodiversity and which habitat units are temporally consistent (e.g. gravel) and others which may display seasonal variability (silt). At present, most national biomonitoring programmes are conducted as multi‐habitat 3‐min kick samples which still enable general riverine health to be assessed. Such assessments will provide reach scale quantification of the ecological community present (Bradley & Ormerod, 2002), however, we advocate that further research should be undertaken at the habitat scale to ensure that the spatial scales we are monitoring capture biodiversity processes that are important for riverine health and reflect the stressor(s) of interest.

### 4.1 Wider implications

Our results highlight the importance of season in structuring both taxonomic and functional macroinvertebrate communities at the habitat level, which has significant implications for scientific research, river management and monitoring. In particular, silt habitats displayed strong seasonal differences in the biodiversity they supported. Silt habitats represent a potentially compositionally unique and highly dynamic habitat which supports functionally comparable communities to other habitats during some seasons and importantly demonstrates greater biodiversity values and differing community structure when sampled in autumn compared to spring. These results suggest that biomonitoring approaches and scientific research investigating the ecological effects of fine sediment deposition may not be fully recognising the ecological functions associated with fine sediment by not incorporating the potential influence of season. For example, it is possible that some species may be indicative of fine sediment stress in some seasons but not in others, with the wider provision of resources and biotic competition affecting the distribution of some taxa between habitats. Silt habitats are likely to be hydraulically unstable and support limited biodiversity in spring. However, as the flow regimes become less variable and potentially biotic interactions increase over the summer months, it is conceivable that a range of taxa can be supported in silt habitats despite the sub‐optimal conditions available. This has important repercussions for the assignment of sensitivity ratings for species and potential quality thresholds of overall biomonitoring metrics and may explain the often equivocal and contrasting findings when trait–sediment relationships are considered. In short, silt habitats represent an important functional niche that clearly has a changing role during different seasons. Gravel and perennial marginal vegetation habitats appear to be more stable over time with the functional composition and richness of these habitats remaining fairly consistent, while sand habitats represented the most impoverished habitat regardless of the season. Despite the common definition of fine sediment being particles <2 mm, sand habitat communities were taxonomically and functionally different to those in silt habitats and as such studies characterising fine sediment pressures should discriminate between sand and silt fractions to accurately characterise the biodiversity supported.

## AUTHOR CONTRIBUTIONS

**Kate L. Mathers:** Conceptualization (lead); data curation (lead); formal analysis (lead); funding acquisition (lead); project administration (lead); visualization (lead); writing – original draft (lead); writing – review and editing (equal). **Patrick D. Armitage:** Conceptualization (supporting); investigation (equal); methodology (equal); writing – review and editing (equal). **Matthew Hill:** Writing – review and editing (equal). **Morwenna McKenzie:** Writing – review and editing (equal). **Isabel Pardo:** Investigation (equal); methodology (equal); writing – review and editing (equal). **Paul J. Wood:** Conceptualization (supporting); writing – review and editing (equal).

## CONFLICT OF INTEREST STATEMENT

The authors declare no conflicts of interest.

## Supporting information


Tables S1–S8

## Data Availability

Data (Mathers et al., 2023) are available from Loughborough University Research Repository: DOI: 10.17028/rd.lboro.24132561.

## References

[ece310564-bib-0001] Angradi, T. R. (1999). Fine sediment and macroinvertebrate assemblages in Appalachian streams: A field experiment with biomonitoring applications. Journal of the North American Benthological Society, 18, 49–66.

[ece310564-bib-0002] Arbizu, P. M. (2019). pairwiseAdonis: Pairwise multilevel comparison using adonis . Retrieved May 5, 2021, from https://github.com/pmartinezarbizu/pairwiseAdonis

[ece310564-bib-0003] Armitage, P. D. (2006). Instream and bankside habitat in rivers. In G. Ziglio , M. Siligardi , & G. Flaim (Eds.), Biological monitoring of rivers. Applications and perspectives (pp. 17–31). Wiley and Sons, Ltd.

[ece310564-bib-0004] Armitage, P. D. , & Cannan, C. E. (2000). Annual changes in summer patterns of habitat distribution and associated macroinvertebrate assemblages. Hydrological Processes, 14, 3161–3179.

[ece310564-bib-0005] Armitage, P. D. , Pardo, I. , & Brown, A. (1995). Temporal constancy of faunal assemblages in ‘habitats’ – Application to management? Archiv für Hydrobiologie, 133, 367–387.

[ece310564-bib-0006] Barnum, T. R. , Weller, D. E. , & Williams, M. (2017). Urbanization reduces and homogenizes trait diversity in stream macroinvertebrate communities. Ecological Applications, 27, 2428–2442. 28872731 10.1002/eap.1619

[ece310564-bib-0007] Beche, L. A. , Mcelravy, E. P. , & Resh, V. H. (2006). Long‐term seasonal variation in the biological traits of benthic‐macroinvertebrates in two Mediterranean‐climate streams in California, USA. Freshwater Biology, 51, 56–75.

[ece310564-bib-0008] Beisel, J. N. , Usseglio‐Polatera, P. , & Moreteau, J. C. (2000). The spatial heterogeneity of a river bottom: A key factor determining macroinvertebrate communities. In M. Jungwirth , S. Muhar , & S. Schmutz (Eds.), Assessing the ecological integrity of running waters (pp. 163–171). Springer.

[ece310564-bib-0009] Belmar, O. , Bruno, D. , Guareschi, S. , Mellado‐Díaz, A. , Millán, A. , & Velasco, J. (2019). Functional responses of aquatic macroinvertebrates to flow regulation are shaped by natural flow intermittence in Mediterranean streams. Freshwater Biology, 64, 1064–1077.

[ece310564-bib-0010] Blöcher, J. R. , Ward, M. R. , Matthaei, C. D. , & Piggott, J. J. (2020). Multiple stressors and stream macroinvertebrate community dynamics: Interactions between fine sediment grain size and flow velocity. Science of the Total Environment, 717, 137070. 32062257 10.1016/j.scitotenv.2020.137070

[ece310564-bib-0011] Bradley, D. C. , & Ormerod, S. J. (2002). Evaluating the precision of kick‐sampling in upland streams for assessments of long‐term change: The effects of sampling effort, habitat and rarity. Archiv für Hydrobiologie, 155, 199–221.

[ece310564-bib-0012] Brunke, M. , Hoffmann, A. , & Pusch, M. (2001). Use of habitat‐specific relationships between flow velocity and river discharge to assess invertebrate minimum flow requirements. Regulated Rivers: Research & Management, 17, 667–676.

[ece310564-bib-0013] Buendia, C. , Gibbins, C. N. , Vericat, D. , Batalla, R. J. , & Douglas, A. (2013). Detecting the structural and functional impacts of fine sediment on stream invertebrates. Ecological Indicators, 25, 184–196.

[ece310564-bib-0014] Burt, T. , Boardman, J. , Foster, I. , & Howden, N. (2016). More rain, less soil: Long‐term changes in rainfall intensity with climate change. Earth Surface Processes and Landforms, 41, 563–566.

[ece310564-bib-0015] Calapez, A. R. , Serra, S. R. , Rivaes, R. , Aguiar, F. C. , & Feio, M. J. (2021). Influence of river regulation and instream habitat on invertebrate assemblage' structure and function. Science of the Total Environment, 794, 148696. 34217076 10.1016/j.scitotenv.2021.148696

[ece310564-bib-0016] Cardoso, P. , Mammola, S. , Rigal, F. , & Carvalho, J. C. (2021). BAT: Biodiversity assessment tools . R package version 2.6.0. Retrieved from https://CRAN.R‐project.org/package=BAT

[ece310564-bib-0017] Cardoso, P. , Rigal, F. , & Carvalho, J. C. (2015). BAT – Biodiversity assessment tools, an R package for the measurement and estimation of alpha and beta taxon, phylogenetic and functional diversity. Methods in Ecology and Evolution, 6, 232–236.

[ece310564-bib-0018] Carlson, P. E. , Johnson, R. K. , & McKie, B. G. (2013). Optimizing stream bioassessment: Habitat, season, and the impacts of land use on benthic macroinvertebrates. Hydrobiologia, 704, 363–373.

[ece310564-bib-0019] Chevene, F. , Doléadec, S. , & Chessel, D. (1994). A fuzzy coding approach for the analysis of long‐term ecological data. Freshwater Biology, 31, 295–309.

[ece310564-bib-0020] Collins, A. L. , & Zhang, Y. (2016). Exceedance of modern ‘background’ fine‐grained sediment delivery to rivers due to current agricultural land use and uptake of water pollution mitigation options across England and Wales. Environmental Science and Policy, 61, 61–73.

[ece310564-bib-0021] Cotton, J. A. , Wharton, G. , Bass, J. A. B. , Heppell, C. M. , & Wotton, R. S. (2006). The effects of seasonal changes to in‐stream vegetation cover on patterns of flow and accumulation of sediment. Geomorphology, 77, 320–334.

[ece310564-bib-0022] Davis, N. G. , Hodson, R. , & Matthaei, C. D. (2022). Long‐term variability in deposited fine sediment and macroinvertebrate communities across different land‐use intensities in a regional set of New Zealand rivers. New Zealand Journal of Marine and Freshwater Research, 56, 191–212.

[ece310564-bib-0023] De Cáceres, M. , Jansen, F. , & Dell, N. (2020). Indicspecies: Relationship between species and groups of sites . R package version 1.7.7. Retrieved from https://cran.r‐project.org/web/packages/indicspecies/

[ece310564-bib-0024] De Cáceres, M. , Legendre, P. , Wiser, S. K. , & Brotons, L. (2012). Using species combinations in indicator value analyses. Methods in Ecology and Evolution, 3, 973–982.

[ece310564-bib-0025] Demars, B. O. , Kemp, J. L. , Friberg, N. , Usseglio‐Polatera, P. , & Harper, D. M. (2012). Linking biotopes to invertebrates in rivers: Biological traits, taxonomic composition and diversity. Ecological Indicators, 23, 301–311.

[ece310564-bib-0026] Descloux, S. , Datry, T. , & Marmonier, P. (2013). Benthic and hyporheic invertebrate assemblages along a gradient of increasing streambed colmation by fine sediment. Aquatic Sciences, 75, 493–507.

[ece310564-bib-0027] Descloux, S. , Datry, T. , & Usseglio‐Polatera, P. (2014). Trait‐based structure of invertebrates along a gradient of sediment colmation: Benthos versus hyporheos responses. Science of the Total Environment, 466, 265–276. 23911840 10.1016/j.scitotenv.2013.06.082

[ece310564-bib-0028] Doretto, A. , Bona, F. , Piano, E. , Zanin, I. , Eandi, A. C. , & Fenoglio, S. (2017). Trophic availability buffers the detrimental effects of clogging in an alpine stream. Science of the Total Environment, 592, 503–511. 28314608 10.1016/j.scitotenv.2017.03.108

[ece310564-bib-0029] Doretto, A. , Piano, E. , Fenoglio, S. , Bona, F. , Crosa, G. , Espa, P. , & Quadroni, S. (2021). Beta‐diversity and stressor specific index reveal patterns of macroinvertebrate community response to sediment flushing. Ecological Indicators, 122, 107256.

[ece310564-bib-0030] dos Reis Oliveira, P. C. , Kraak, M. H. , van der Geest, H. G. , Naranjo, S. , & Verdonschot, P. F. (2018). Sediment composition mediated land use effects on lowland streams ecosystems. Science of the Total Environment, 631, 459–468. 29529434 10.1016/j.scitotenv.2018.03.010

[ece310564-bib-0031] Dufrêne, M. , & Legendre, P. (1997). Species assemblages and indicator species: The need for a flexible asymmetrical approach. Ecological Monographs, 67, 345–366.

[ece310564-bib-0032] England, J. , & Wilkes, M. A. (2018). Does river restoration work? Taxonomic and functional trajectories at two restoration schemes. Science of the Total Environment, 618, 961–970. 29126643 10.1016/j.scitotenv.2017.09.014

[ece310564-bib-0033] Ferreiro, N. , Giorgi, A. , & Feijoó, C. (2013). Effects of macrophyte architecture and leaf shape complexity on structural parameters of the epiphytic algal community in a Pampean stream. Aquatic Ecology, 47, 389–401.

[ece310564-bib-0034] Gayraud, S. , Statzner, B. , Bady, P. , Haybachp, A. , Schöll, F. , Usseglio‐ Polatera, P. , & Bacchi, M. (2003). Invertebrate traits for the biomonitoring of large European rivers: An initial assessment of alternative metrics. Freshwater Biology, 48, 2045–2064.

[ece310564-bib-0035] Guareschi, S. , Laini, A. , England, J. , Johns, T. , Winter, M. , & Wood, P. J. (2021). Invasive species influence macroinvertebrate biomonitoring tools and functional diversity in British rivers. Journal of Applied Ecology, 58, 135–147.

[ece310564-bib-0036] Gunn, R. J. M. (1985). The biology of *Brachycentrus subnubilus* Curtis (Trichoptera) in the River Frome, Dorset. Hydrobiologia, 120, 133–1401.

[ece310564-bib-0037] Harper, D. , & Everard, M. (1998). Why should the habitat‐level approach underpin holistic river survey and management? Aquatic Conservation: Marine and Freshwater Ecosystems, 8, 395–413.

[ece310564-bib-0038] Helms, B. S. , Schoonover, J. E. , & Feminella, J. W. (2009). Seasonal variability of landuse impacts on macroinvertebrate assemblages in streams of western Georgia, USA. Journal of the North American Benthological Society, 28, 991–1006.

[ece310564-bib-0039] Heywood, M. J. T. , & Walling, D. E. (2003). Suspended sediment fluxes in chalk streams in the Hampshire Avon catchment, UK. Hydrobiologia, 494, 111–117.

[ece310564-bib-0040] Hynes, H. B. N. (1972). The ecology of running waters (p. 555). University of Toronto Press.

[ece310564-bib-0041] Jensen, M. R. , Egelyng Sigsgaard, E. , Agersnap, S. , Jessen Rasmussen, J. , Baattrup‐Pedersen, A. , Wiberg‐Larsen, P. , & Francis Thomsen, P. (2021). Seasonal turnover in community composition of stream‐associated macroinvertebrates inferred from freshwater environmental DNA metabarcoding. Environmental DNA, 3, 861–876.

[ece310564-bib-0042] Johnson, R. C. , Carreiro, M. M. , Jin, H. S. , & Jack, J. D. (2012). Within‐year temporal variation and life‐cycle seasonality affect stream macroinvertebrate community structure and biotic metrics. Ecological Indicators, 13, 206–214.

[ece310564-bib-0043] Juvigny‐Khenafou, N. P. , Piggott, J. J. , Atkinson, D. , Zhang, Y. , Macaulay, S. J. , Wu, N. , & Matthaei, C. D. (2021). Impacts of multiple anthropogenic stressors on stream macroinvertebrate community composition and functional diversity. Ecology and Evolution, 11, 133–152. 33437419 10.1002/ece3.6979 PMC7790656

[ece310564-bib-0044] Kaller, M. D. , & Hartman, K. J. (2004). Evidence of a threshold level of fine sediment accumulation for altering benthic macroinvertebrate communities. Hydrobiologia, 518, 95–104.

[ece310564-bib-0045] Kemp, J. L. , Harper, D. M. , & Crosa, G. A. (2000). The habitat‐scale ecohydraulics of rivers. Ecological Engineering, 16, 17–29.

[ece310564-bib-0046] Laliberté, E. , Legendre, P. , & Shipley, B. (2014). FD: Measuring functional diversity from multiple traits, and other tools for functional ecology . R package version 1.0‐12. 10.1890/08-2244.1 20380219

[ece310564-bib-0047] Larsen, S. , Vaughan, I. P. , & Ormerod, S. J. (2009). Scale‐dependent effects of fine sediments on temperate headwater invertebrates. Freshwater Biology, 54, 203–219.

[ece310564-bib-0048] Lenth, R. , Buerkner, P. , Herve, M. , Love, J. , Riebl, H. , & Singmann, H. (2020). emmeans . Retrieved from https://CRAN.R project.org/package=emmeans

[ece310564-bib-0049] Linke, S. , Bailey, R. C. , & Schwindt, J. (1999). Temporal variability of stream bioassessments using benthic macroinvertebrates. Freshwater Biology, 42, 575–584.

[ece310564-bib-0050] Lürig, M. D. , Best, R. J. , Dakos, V. , & Matthews, B. (2021). Submerged macrophytes affect the temporal variability of aquatic ecosystems. Freshwater Biology, 66, 421–435.

[ece310564-bib-0051] Lytle, D. A. , & Poff, N. L. (2004). Adaptation to natural flow regimes. Trends in Ecology & Evolution, 19, 94–100. 16701235 10.1016/j.tree.2003.10.002

[ece310564-bib-0052] Magliozzi, C. , Meyer, A. , Usseglio‐Polatera, P. , Robertson, A. , & Grabowski, R. C. (2020). Investigating invertebrate biodiversity around large wood: Taxonomic vs functional metrics. Aquatic Sciences, 82, 1–13.32489242

[ece310564-bib-0053] Martins, I. , Castro, D. M. , Macedo, D. R. , Hughes, R. M. , & Callisto, M. (2021). Anthropogenic impacts influence the functional traits of Chironomidae (Diptera) assemblages in a neotropical savanna river basin. Aquatic Ecology, 55, 1081–1095.

[ece310564-bib-0054] Mason, N. W. , Mouillot, D. , Lee, W. G. , & Wilson, J. B. (2005). Functional richness, functional evenness and functional divergence: The primary components of functional diversity. Oikos, 111, 112–118.

[ece310564-bib-0055] Mathers, K. , Armitage, P. , Hill, M. , McKenzie, M. , Pardo, I. , & Wood, P. (2023). Seasonal habitat macroinvertebrate data from Mill Stream, Dorset, UK. Loughborough University Dataset. 10.17028/rd.lboro.24132561

[ece310564-bib-0056] Mathers, K. L. , Doretto, A. , Fenoglio, S. , Hill, M. J. , & Wood, P. J. (2022). Temporal effects of fine sediment deposition on benthic macroinvertebrate community structure, function and biodiversity likely reflects landscape setting. Science of the Total Environment, 829, 154612. 35307447 10.1016/j.scitotenv.2022.154612

[ece310564-bib-0057] Mathers, K. L. , Hill, M. J. , Wood, C. D. , & Wood, P. J. (2019). The role of fine sediment characteristics and body size on the vertical movement of a freshwater amphipod. Freshwater Biology, 64, 152–163.

[ece310564-bib-0058] Mathers, K. L. , Rice, S. P. , & Wood, P. J. (2017). Temporal effects of enhanced fine sediment loading on macroinvertebrate community structure and functional traits. Science of the Total Environment, 599, 513–522. 28482308 10.1016/j.scitotenv.2017.04.096

[ece310564-bib-0059] Mathers, K. L. , White, J. C. , Guareschi, S. , Hill, M. J. , Heino, J. , & Chadd, R. (2020). Invasive crayfish alter the long‐term functional biodiversity of lotic macroinvertebrate communities. Functional Ecology, 34, 2350–2361.

[ece310564-bib-0060] Matthaei, C. D. , Weller, F. , Kelly, D. W. , & Townsend, C. R. (2006). Impacts of fine sediment addition to tussock, pasture, dairy and deer farming streams in New Zealand. Freshwater Biology, 51, 2154–2172.

[ece310564-bib-0061] Mazor, R. D. , Purcell, A. H. , & Resh, V. H. (2009). Long‐term variability in bioassessments: A twenty‐year study from two northern California streams. Environmental Management, 43, 1269–1286. 19381714 10.1007/s00267-009-9294-8 PMC2691804

[ece310564-bib-0062] McKenzie, M. , England, J. , Foster, I. D. , & Wilkes, M. A. (2022). Abiotic predictors of fine sediment accumulation in lowland rivers. International Journal of Sediment Research, 37, 128–137.

[ece310564-bib-0063] Mouillot, D. , Graham, N. A. , Villéger, S. , Mason, N. W. , & Bellwood, D. R. (2013). A functional approach reveals community responses to disturbances. Trends in Ecology & Evolution, 28, 167–177. 23141923 10.1016/j.tree.2012.10.004

[ece310564-bib-0064] Newson, M. D. , & Newson, C. L. (2000). Geomorphology, ecology and river channel habitat: Mesoscale approaches to basin‐scale challenges. Progress in Physical Geography, 24, 195–217.

[ece310564-bib-0065] Oksanen, J. , Blanchet, F. G. , Friendly, M. , Kindt, R. , Legendre, P. , McGlinn, D. , Minchin, P. R. , O'Hara, R. B. , Simpson, G. L. , Solymos, P. , Henry, M. , Stevens, H. , Szoecs, E. , & Wagner, H. (2019). Vegan: Community ecology package . R package version 2.5‐6.

[ece310564-bib-0066] Ormerod, S. J. (1987). The influences of habitat and seasonal sampling regimes on the ordination and classification of macroinvertebrate assemblages in the catchment of the River Wye, Wales. Hydrobiologia, 150, 143–151.

[ece310564-bib-0067] Pardo, I. , & Armitage, P. D. (1997). Species assemblages as descriptors of habitats. Hydrobiologia, 344, 111–128.

[ece310564-bib-0068] Power, M. E. , Stout, R. J. , Cushing, C. E. , Harper, P. P. , Hauer, F. R. , Matthews, W. J. , Moyle, P. B. , Statzner, B. , Irene, R. W. D. B. , & De Badgen, W. (1988). Biotic and abiotic controls in river and stream communities. Journal of the North American Benthological Society, 7(4), 456–479.

[ece310564-bib-0069] R Development Core Team . (2022). R: A language and environment for statistical computing. R Foundation for Statistical Computing.

[ece310564-bib-0070] Richards, C. , Host, G. E. , & Arthur, J. W. (1993). Identification of predominant environmental factors structuring stream macroinvertebrate communities within a large agricultural catchment. Freshwater Biology, 29, 285–294.

[ece310564-bib-0071] Salmaso, F. , Espa, P. , Crosa, G. , & Quadroni, S. (2021). Impacts of fine sediment input on river macroinvertebrates: The role of the abiotic characteristics at habitat scale. Hydrobiologia, 848, 4189–4209.

[ece310564-bib-0072] Schliep, K. (2011). Phangorn: Phylogenetic analysis in R. Bioinformatics, 27, 592–593. 21169378 10.1093/bioinformatics/btq706 PMC3035803

[ece310564-bib-0073] Schmera, D. , Erős, T. , & Heino, J. (2013). Habitat filtering determines spatial variation of macroinvertebrate community traits in northern headwater streams. Community Ecology, 14, 77–88.

[ece310564-bib-0074] Sherriff, S. C. , Rowan, J. S. , Fenton, O. , & Jordan, P. (2018). Sediment fingerprinting as a tool to identify temporal and spatial variability of sediment sources and transport pathways in agricultural catchments. Agriculture, Ecosystems & Environment, 267, 188–200.

[ece310564-bib-0075] Sofi, M. S. , Bhat, S. U. , Rashid, I. , & Kuniyal, J. C. (2020). The natural flow regime: A master variable for maintaining river ecosystem health. Ecohydrology, 13, e2247.

[ece310564-bib-0076] Southwood, T. R. (1977). Habitat, the templet for ecological strategies? Journal of Animal Ecology, 46, 337–365.

[ece310564-bib-0077] Tachet, H. , Bournaud, M. , Richoux, P. , & Usseglio‐Polatera, P. (2010). Invertébrés d'eau douce: Systématique, biologie, écologie. CNRS Editions.

[ece310564-bib-0078] Thioulouse, J. , Dray, S. , Dufour, A. , Siberchicot, A. , Jombart, T. , & Pavoine, S. (2018). Multivariate analysis of ecological data with ade4. Springer.

[ece310564-bib-0079] Turley, M. D. , Bilotta, G. S. , Chadd, R. P. , Extence, C. A. , Brazier, R. E. , Burnside, N. G. , & Pickwell, A. G. (2016). A sediment‐specific family‐level biomonitoring tool to identify the impacts of fine sediment in temperate rivers and streams. Ecological Indicators, 70, 151–165.

[ece310564-bib-0080] Villéger, S. , Mason, N. W. , & Mouillot, D. (2008). New multidimensional functional diversity indices for a multifaceted framework in functional ecology. Ecology, 89, 2290–2301. 18724739 10.1890/07-1206.1

[ece310564-bib-0081] White, J. C. , Fornaroli, R. , Hill, M. J. , Hannah, D. M. , House, A. , Colley, I. , Perkins, M. , & Wood, P. J. (2021). Long‐term river invertebrate community responses to groundwater and surface water management operations. Water Research, 189, 116651. 33248332 10.1016/j.watres.2020.116651

[ece310564-bib-0082] White, J. C. , Hill, M. J. , Bickerton, M. A. , & Wood, P. J. (2017). Macroinvertebrate taxonomic and functional trait compositions within lotic habitats affected by river restoration practices. Environmental Management, 60, 513–525. 28516312 10.1007/s00267-017-0889-1 PMC5544791

[ece310564-bib-0083] Wilkes, M. A. , Mckenzie, M. , Murphy, J. F. , & Chadd, R. P. (2017). Assessing the mechanistic basis for fine sediment biomonitoring: Inconsistencies among the literature, traits and indices. River Research and Applications, 33, 1618–1629.

[ece310564-bib-0084] Wolters, J. W. , Verdonschot, R. , Schoelynck, J. , Verdonschot, P. F. , & Meire, P. (2018). The role of macrophyte structural complexity and water flow velocity in determining the epiphytic macroinvertebrate community composition in a lowland stream. Hydrobiologia, 806, 157–173.

[ece310564-bib-0085] Worrall, T. P. , Dunbar, M. J. , Extence, C. A. , Laize, C. L. , Monk, W. A. , & Wood, P. J. (2014). The identification of hydrological indices for the characterization of macroinvertebrate community response to flow regime variability. Hydrological Sciences Journal, 59, 645–658.

