# Supplementary material for: Seasonal variability of lotic macroinvertebrate communities at the habitat scale demonstrates the value of discriminating fine sediment fractions in ecological assessments
Source: Ecol Evol. 2023 Sep 29;13(10):e10564. doi: 10.1002/ece3.10564 (PMC10541294; doi:10.1002/ece3.10564)
Supplement: Supplementary file 1 — Tables S1–S8 [file ECE3-13-e10564-s001.pptx]

## Slide 1
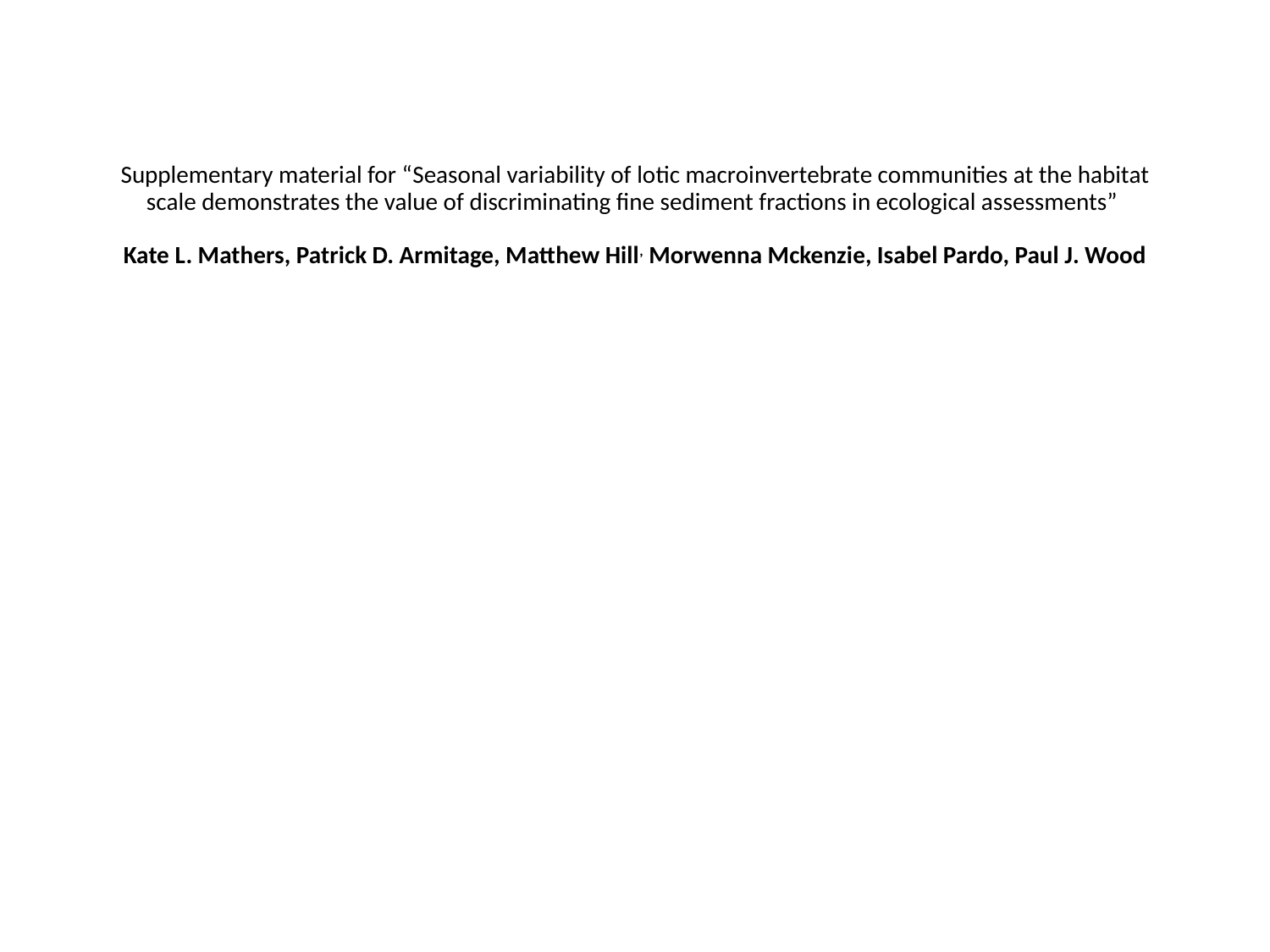

# Supplementary material for “Seasonal variability of lotic macroinvertebrate communities at the habitat scale demonstrates the value of discriminating fine sediment fractions in ecological assessments” Kate L. Mathers, Patrick D. Armitage, Matthew Hill, Morwenna Mckenzie, Isabel Pardo, Paul J. Wood

## Slide 2
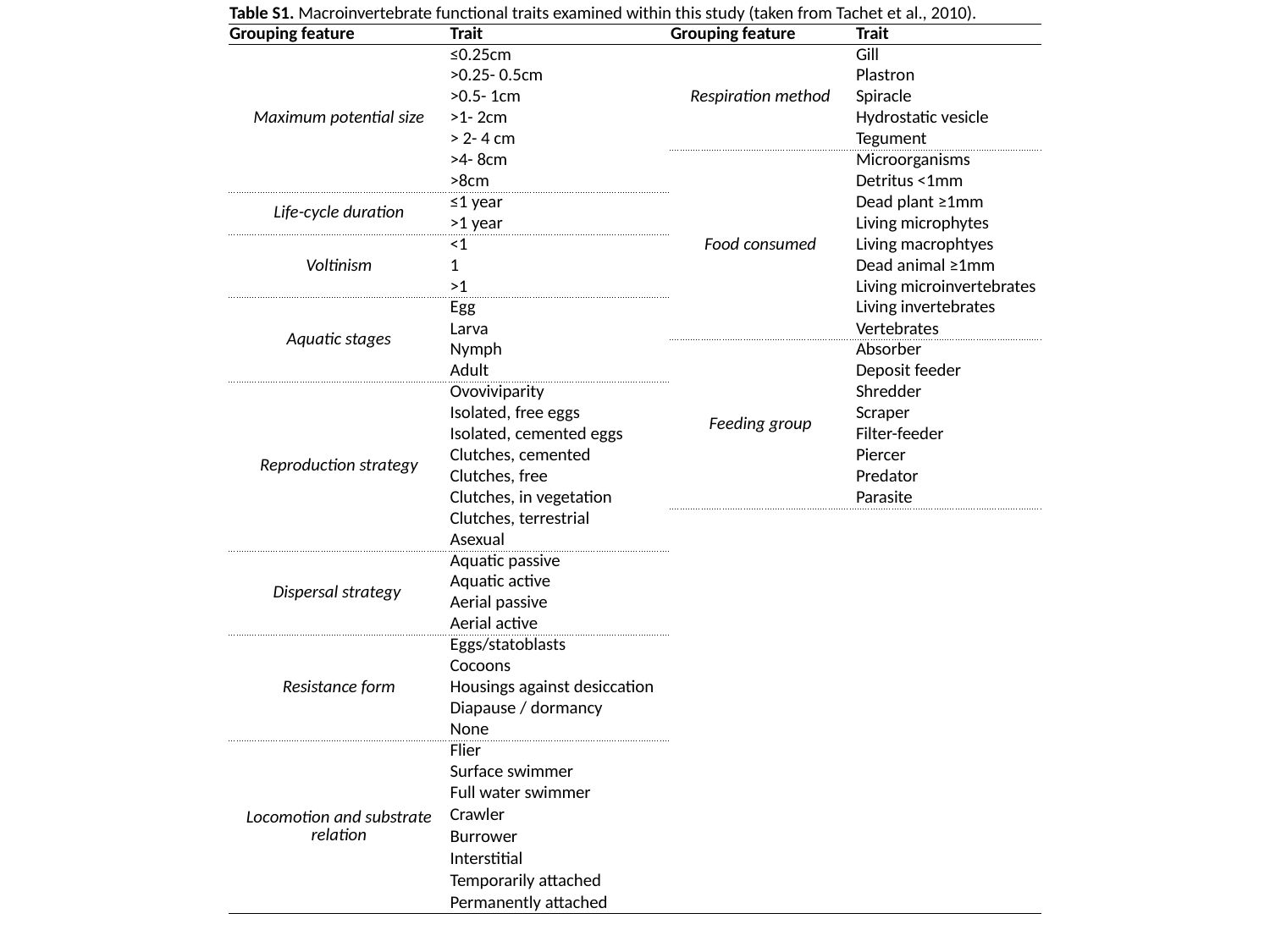

| Table S1. Macroinvertebrate functional traits examined within this study (taken from Tachet et al., 2010). | | | |
| --- | --- | --- | --- |
| Grouping feature | Trait | Grouping feature | Trait |
| Maximum potential size | ≤0.25cm | Respiration method | Gill |
| | >0.25- 0.5cm | | Plastron |
| | >0.5- 1cm | | Spiracle |
| | >1- 2cm | | Hydrostatic vesicle |
| | > 2- 4 cm | | Tegument |
| | >4- 8cm | Food consumed | Microorganisms |
| | >8cm | | Detritus <1mm |
| Life-cycle duration | ≤1 year | | Dead plant ≥1mm |
| | >1 year | | Living microphytes |
| Voltinism | <1 | | Living macrophtyes |
| | 1 | | Dead animal ≥1mm |
| | >1 | | Living microinvertebrates |
| Aquatic stages | Egg | | Living invertebrates |
| | Larva | | Vertebrates |
| | Nymph | Feeding group | Absorber |
| | Adult | | Deposit feeder |
| Reproduction strategy | Ovoviviparity | | Shredder |
| | Isolated, free eggs | | Scraper |
| | Isolated, cemented eggs | | Filter-feeder |
| | Clutches, cemented | | Piercer |
| | Clutches, free | | Predator |
| | Clutches, in vegetation | | Parasite |
| | Clutches, terrestrial | | |
| | Asexual | | |
| Dispersal strategy | Aquatic passive | | |
| | Aquatic active | | |
| | Aerial passive | | |
| | Aerial active | | |
| Resistance form | Eggs/statoblasts | | |
| | Cocoons | | |
| | Housings against desiccation | | |
| | Diapause / dormancy | | |
| | None | | |
| Locomotion and substrate relation | Flier | | |
| | Surface swimmer | | |
| | Full water swimmer | | |
| | Crawler | | |
| | Burrower | | |
| | Interstitial | | |
| | Temporarily attached | | |
| | Permanently attached | | |

## Slide 3
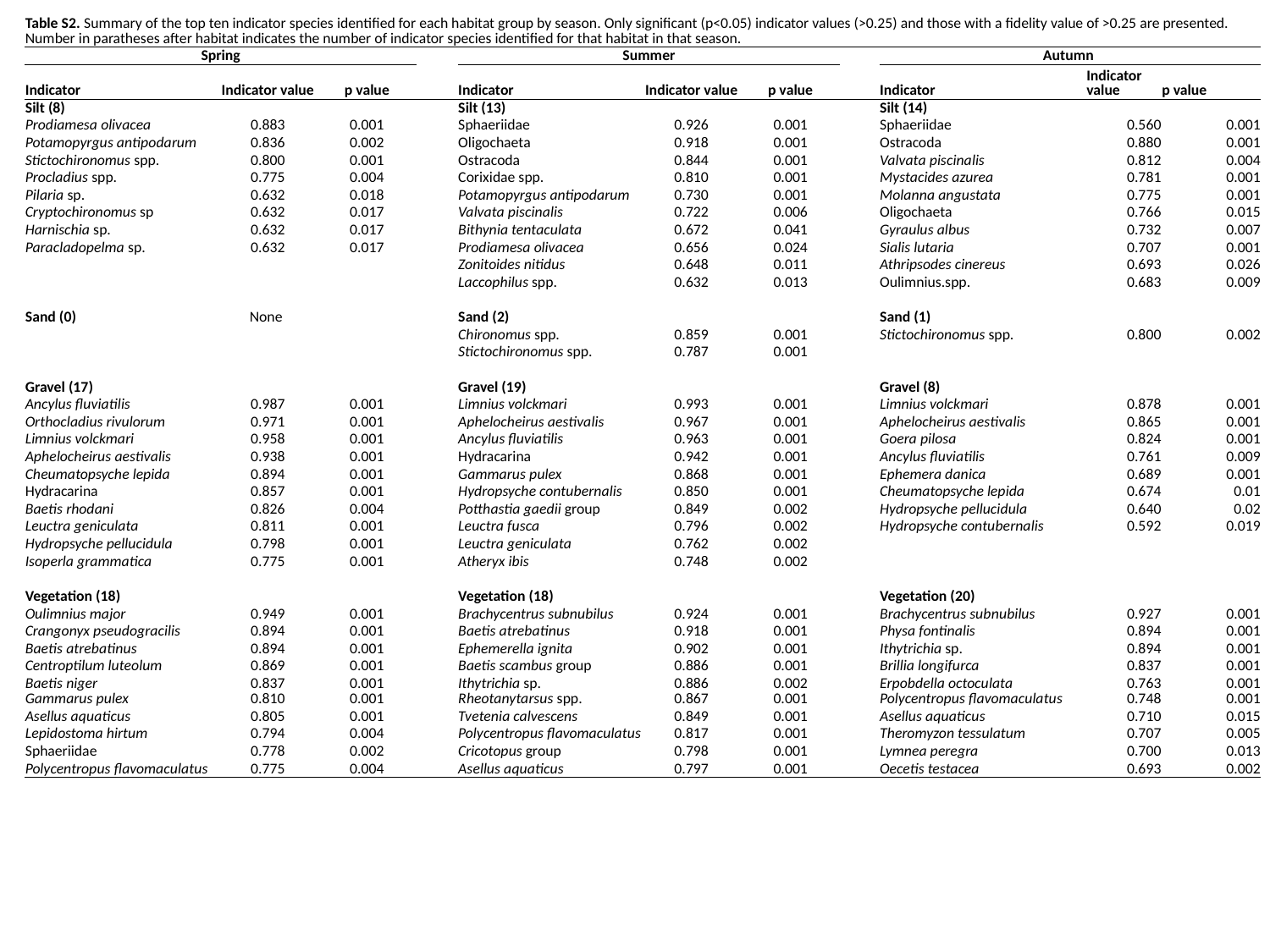

| Table S2. Summary of the top ten indicator species identified for each habitat group by season. Only significant (p<0.05) indicator values (>0.25) and those with a fidelity value of >0.25 are presented. Number in paratheses after habitat indicates the number of indicator species identified for that habitat in that season. | | | | | | | | | | |
| --- | --- | --- | --- | --- | --- | --- | --- | --- | --- | --- |
| Spring | | | | Summer | | | | Autumn | | |
| Indicator | Indicator value | p value | | Indicator | Indicator value | p value | | Indicator | Indicator value | p value |
| Silt (8) | | | | Silt (13) | | | | Silt (14) | | |
| Prodiamesa olivacea | 0.883 | 0.001 | | Sphaeriidae | 0.926 | 0.001 | | Sphaeriidae | 0.560 | 0.001 |
| Potamopyrgus antipodarum | 0.836 | 0.002 | | Oligochaeta | 0.918 | 0.001 | | Ostracoda | 0.880 | 0.001 |
| Stictochironomus spp. | 0.800 | 0.001 | | Ostracoda | 0.844 | 0.001 | | Valvata piscinalis | 0.812 | 0.004 |
| Procladius spp. | 0.775 | 0.004 | | Corixidae spp. | 0.810 | 0.001 | | Mystacides azurea | 0.781 | 0.001 |
| Pilaria sp. | 0.632 | 0.018 | | Potamopyrgus antipodarum | 0.730 | 0.001 | | Molanna angustata | 0.775 | 0.001 |
| Cryptochironomus sp | 0.632 | 0.017 | | Valvata piscinalis | 0.722 | 0.006 | | Oligochaeta | 0.766 | 0.015 |
| Harnischia sp. | 0.632 | 0.017 | | Bithynia tentaculata | 0.672 | 0.041 | | Gyraulus albus | 0.732 | 0.007 |
| Paracladopelma sp. | 0.632 | 0.017 | | Prodiamesa olivacea | 0.656 | 0.024 | | Sialis lutaria | 0.707 | 0.001 |
| | | | | Zonitoides nitidus | 0.648 | 0.011 | | Athripsodes cinereus | 0.693 | 0.026 |
| | | | | Laccophilus spp. | 0.632 | 0.013 | | Oulimnius.spp. | 0.683 | 0.009 |
| | | | | | | | | | | |
| Sand (0) | None | | | Sand (2) | | | | Sand (1) | | |
| | | | | Chironomus spp. | 0.859 | 0.001 | | Stictochironomus spp. | 0.800 | 0.002 |
| | | | | Stictochironomus spp. | 0.787 | 0.001 | | | | |
| | | | | | | | | | | |
| Gravel (17) | | | | Gravel (19) | | | | Gravel (8) | | |
| Ancylus fluviatilis | 0.987 | 0.001 | | Limnius volckmari | 0.993 | 0.001 | | Limnius volckmari | 0.878 | 0.001 |
| Orthocladius rivulorum | 0.971 | 0.001 | | Aphelocheirus aestivalis | 0.967 | 0.001 | | Aphelocheirus aestivalis | 0.865 | 0.001 |
| Limnius volckmari | 0.958 | 0.001 | | Ancylus fluviatilis | 0.963 | 0.001 | | Goera pilosa | 0.824 | 0.001 |
| Aphelocheirus aestivalis | 0.938 | 0.001 | | Hydracarina | 0.942 | 0.001 | | Ancylus fluviatilis | 0.761 | 0.009 |
| Cheumatopsyche lepida | 0.894 | 0.001 | | Gammarus pulex | 0.868 | 0.001 | | Ephemera danica | 0.689 | 0.001 |
| Hydracarina | 0.857 | 0.001 | | Hydropsyche contubernalis | 0.850 | 0.001 | | Cheumatopsyche lepida | 0.674 | 0.01 |
| Baetis rhodani | 0.826 | 0.004 | | Potthastia gaedii group | 0.849 | 0.002 | | Hydropsyche pellucidula | 0.640 | 0.02 |
| Leuctra geniculata | 0.811 | 0.001 | | Leuctra fusca | 0.796 | 0.002 | | Hydropsyche contubernalis | 0.592 | 0.019 |
| Hydropsyche pellucidula | 0.798 | 0.001 | | Leuctra geniculata | 0.762 | 0.002 | | | | |
| Isoperla grammatica | 0.775 | 0.001 | | Atheryx ibis | 0.748 | 0.002 | | | | |
| | | | | | | | | | | |
| Vegetation (18) | | | | Vegetation (18) | | | | Vegetation (20) | | |
| Oulimnius major | 0.949 | 0.001 | | Brachycentrus subnubilus | 0.924 | 0.001 | | Brachycentrus subnubilus | 0.927 | 0.001 |
| Crangonyx pseudogracilis | 0.894 | 0.001 | | Baetis atrebatinus | 0.918 | 0.001 | | Physa fontinalis | 0.894 | 0.001 |
| Baetis atrebatinus | 0.894 | 0.001 | | Ephemerella ignita | 0.902 | 0.001 | | Ithytrichia sp. | 0.894 | 0.001 |
| Centroptilum luteolum | 0.869 | 0.001 | | Baetis scambus group | 0.886 | 0.001 | | Brillia longifurca | 0.837 | 0.001 |
| Baetis niger | 0.837 | 0.001 | | Ithytrichia sp. | 0.886 | 0.002 | | Erpobdella octoculata | 0.763 | 0.001 |
| Gammarus pulex | 0.810 | 0.001 | | Rheotanytarsus spp. | 0.867 | 0.001 | | Polycentropus flavomaculatus | 0.748 | 0.001 |
| Asellus aquaticus | 0.805 | 0.001 | | Tvetenia calvescens | 0.849 | 0.001 | | Asellus aquaticus | 0.710 | 0.015 |
| Lepidostoma hirtum | 0.794 | 0.004 | | Polycentropus flavomaculatus | 0.817 | 0.001 | | Theromyzon tessulatum | 0.707 | 0.005 |
| Sphaeriidae | 0.778 | 0.002 | | Cricotopus group | 0.798 | 0.001 | | Lymnea peregra | 0.700 | 0.013 |
| Polycentropus flavomaculatus | 0.775 | 0.004 | | Asellus aquaticus | 0.797 | 0.001 | | Oecetis testacea | 0.693 | 0.002 |

## Slide 4
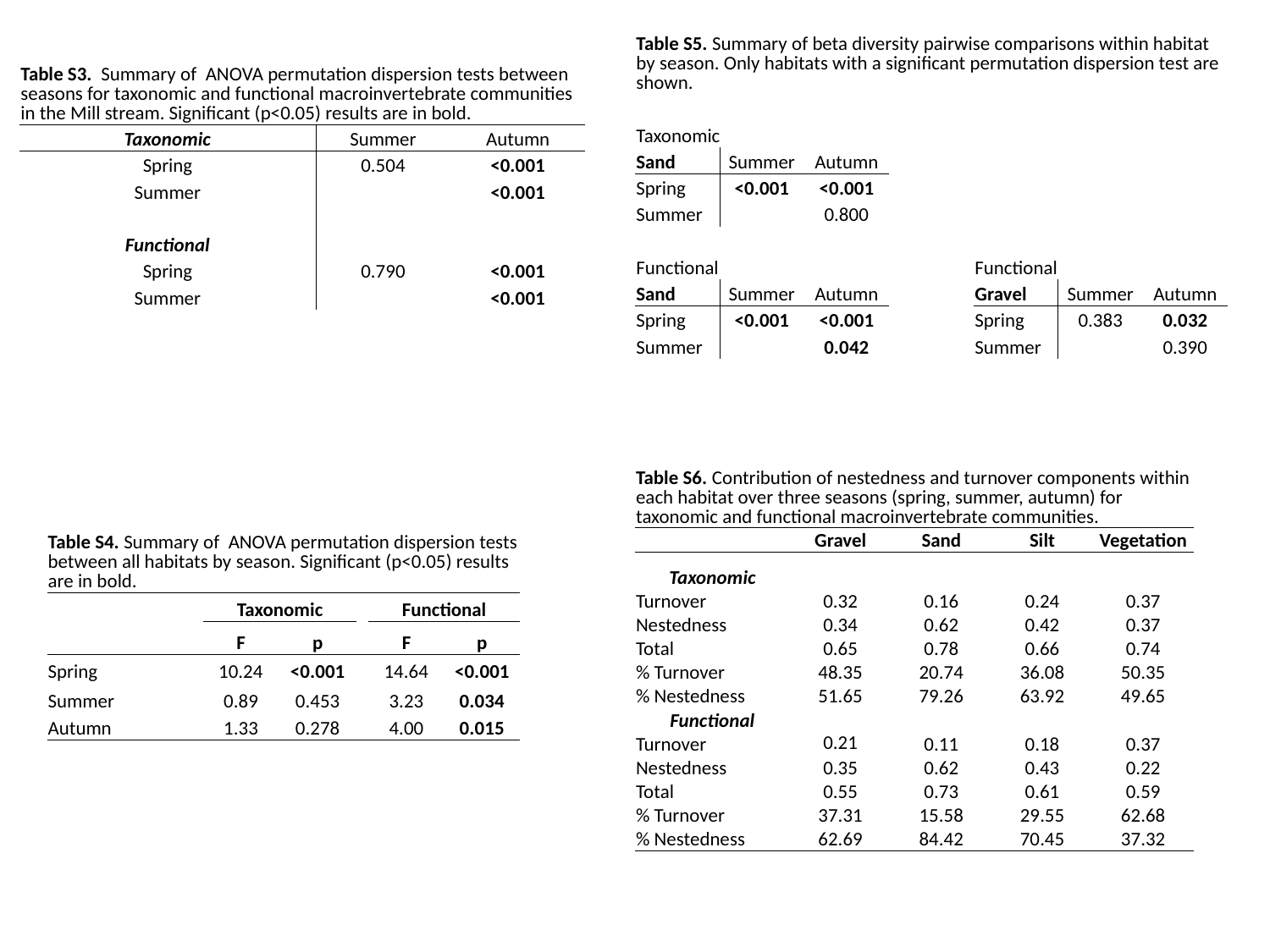

| Table S5. Summary of beta diversity pairwise comparisons within habitat by season. Only habitats with a significant permutation dispersion test are shown. | | | | | | |
| --- | --- | --- | --- | --- | --- | --- |
| | | | | | | |
| Taxonomic | | | | | | |
| Sand | Summer | Autumn | | | | |
| Spring | <0.001 | <0.001 | | | | |
| Summer | | 0.800 | | | | |
| | | | | | | |
| Functional | | | | Functional | | |
| Sand | Summer | Autumn | | Gravel | Summer | Autumn |
| Spring | <0.001 | <0.001 | | Spring | 0.383 | 0.032 |
| Summer | | 0.042 | | Summer | | 0.390 |
| Table S3. Summary of ANOVA permutation dispersion tests between seasons for taxonomic and functional macroinvertebrate communities in the Mill stream. Significant (p<0.05) results are in bold. | | |
| --- | --- | --- |
| Taxonomic | Summer | Autumn |
| Spring | 0.504 | <0.001 |
| Summer | | <0.001 |
| | | |
| Functional | | |
| Spring | 0.790 | <0.001 |
| Summer | | <0.001 |
| Table S6. Contribution of nestedness and turnover components within each habitat over three seasons (spring, summer, autumn) for taxonomic and functional macroinvertebrate communities. | | | | |
| --- | --- | --- | --- | --- |
| | Gravel | Sand | Silt | Vegetation |
| Taxonomic | | | | |
| Turnover | 0.32 | 0.16 | 0.24 | 0.37 |
| Nestedness | 0.34 | 0.62 | 0.42 | 0.37 |
| Total | 0.65 | 0.78 | 0.66 | 0.74 |
| % Turnover | 48.35 | 20.74 | 36.08 | 50.35 |
| % Nestedness | 51.65 | 79.26 | 63.92 | 49.65 |
| Functional | | | | |
| Turnover | 0.21 | 0.11 | 0.18 | 0.37 |
| Nestedness | 0.35 | 0.62 | 0.43 | 0.22 |
| Total | 0.55 | 0.73 | 0.61 | 0.59 |
| % Turnover | 37.31 | 15.58 | 29.55 | 62.68 |
| % Nestedness | 62.69 | 84.42 | 70.45 | 37.32 |
| Table S4. Summary of ANOVA permutation dispersion tests between all habitats by season. Significant (p<0.05) results are in bold. | | | | | |
| --- | --- | --- | --- | --- | --- |
| | Taxonomic | | | Functional | |
| | F | p | | F | p |
| Spring | 10.24 | <0.001 | | 14.64 | <0.001 |
| Summer | 0.89 | 0.453 | | 3.23 | 0.034 |
| Autumn | 1.33 | 0.278 | | 4.00 | 0.015 |

## Slide 5
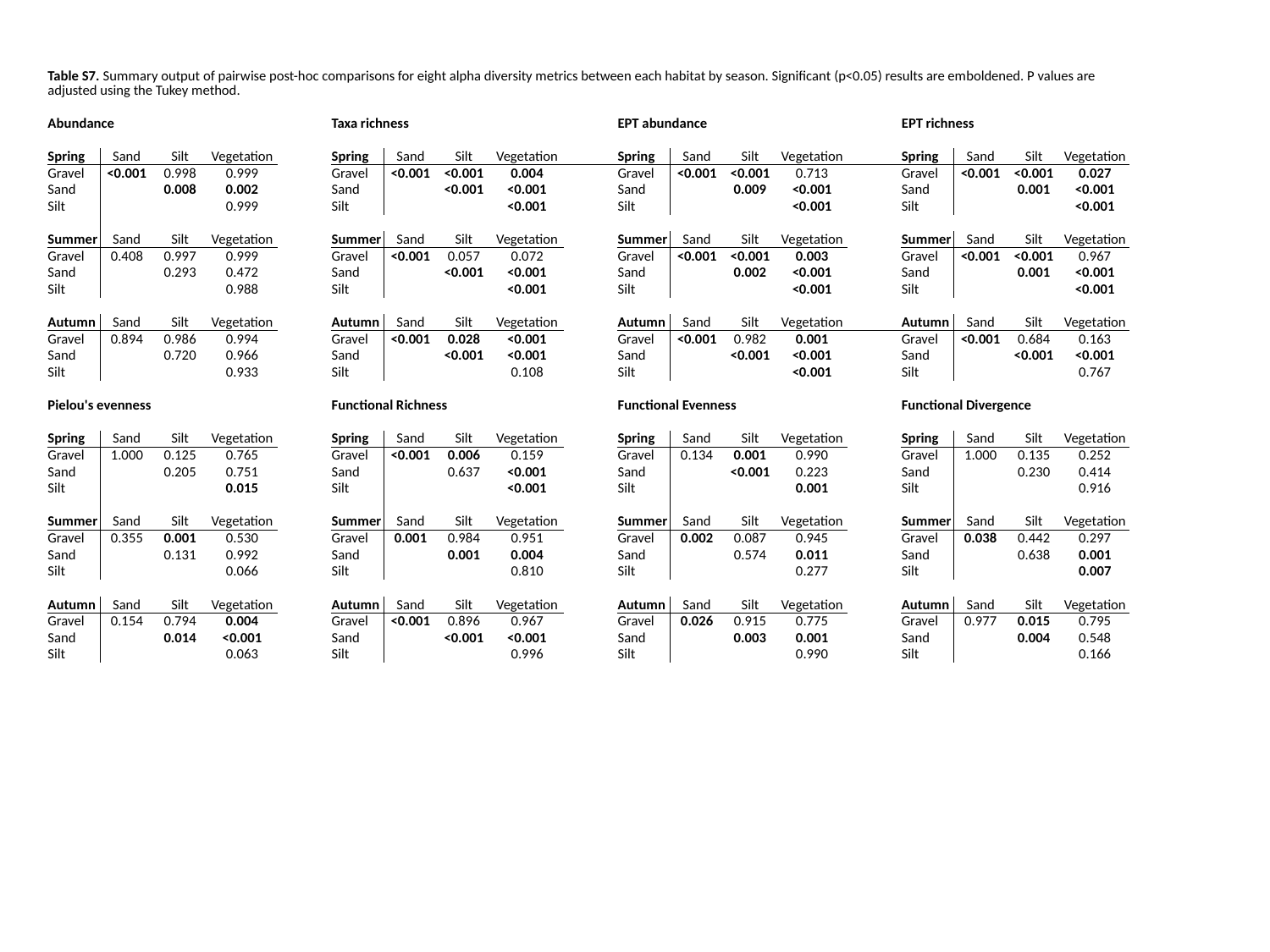

| Table S7. Summary output of pairwise post-hoc comparisons for eight alpha diversity metrics between each habitat by season. Significant (p<0.05) results are emboldened. P values are adjusted using the Tukey method. | | | | | | | | | | | | | | | | | | |
| --- | --- | --- | --- | --- | --- | --- | --- | --- | --- | --- | --- | --- | --- | --- | --- | --- | --- | --- |
| | | | | | | | | | | | | | | | | | | |
| Abundance | | | | | Taxa richness | | | | | EPT abundance | | | | | EPT richness | | | |
| | | | | | | | | | | | | | | | | | | |
| Spring | Sand | Silt | Vegetation | | Spring | Sand | Silt | Vegetation | | Spring | Sand | Silt | Vegetation | | Spring | Sand | Silt | Vegetation |
| Gravel | <0.001 | 0.998 | 0.999 | | Gravel | <0.001 | <0.001 | 0.004 | | Gravel | <0.001 | <0.001 | 0.713 | | Gravel | <0.001 | <0.001 | 0.027 |
| Sand | | 0.008 | 0.002 | | Sand | | <0.001 | <0.001 | | Sand | | 0.009 | <0.001 | | Sand | | 0.001 | <0.001 |
| Silt | | | 0.999 | | Silt | | | <0.001 | | Silt | | | <0.001 | | Silt | | | <0.001 |
| | | | | | | | | | | | | | | | | | | |
| Summer | Sand | Silt | Vegetation | | Summer | Sand | Silt | Vegetation | | Summer | Sand | Silt | Vegetation | | Summer | Sand | Silt | Vegetation |
| Gravel | 0.408 | 0.997 | 0.999 | | Gravel | <0.001 | 0.057 | 0.072 | | Gravel | <0.001 | <0.001 | 0.003 | | Gravel | <0.001 | <0.001 | 0.967 |
| Sand | | 0.293 | 0.472 | | Sand | | <0.001 | <0.001 | | Sand | | 0.002 | <0.001 | | Sand | | 0.001 | <0.001 |
| Silt | | | 0.988 | | Silt | | | <0.001 | | Silt | | | <0.001 | | Silt | | | <0.001 |
| | | | | | | | | | | | | | | | | | | |
| Autumn | Sand | Silt | Vegetation | | Autumn | Sand | Silt | Vegetation | | Autumn | Sand | Silt | Vegetation | | Autumn | Sand | Silt | Vegetation |
| Gravel | 0.894 | 0.986 | 0.994 | | Gravel | <0.001 | 0.028 | <0.001 | | Gravel | <0.001 | 0.982 | 0.001 | | Gravel | <0.001 | 0.684 | 0.163 |
| Sand | | 0.720 | 0.966 | | Sand | | <0.001 | <0.001 | | Sand | | <0.001 | <0.001 | | Sand | | <0.001 | <0.001 |
| Silt | | | 0.933 | | Silt | | | 0.108 | | Silt | | | <0.001 | | Silt | | | 0.767 |
| | | | | | | | | | | | | | | | | | | |
| Pielou's evenness | | | | | Functional Richness | | | | | Functional Evenness | | | | | Functional Divergence | | | |
| | | | | | | | | | | | | | | | | | | |
| Spring | Sand | Silt | Vegetation | | Spring | Sand | Silt | Vegetation | | Spring | Sand | Silt | Vegetation | | Spring | Sand | Silt | Vegetation |
| Gravel | 1.000 | 0.125 | 0.765 | | Gravel | <0.001 | 0.006 | 0.159 | | Gravel | 0.134 | 0.001 | 0.990 | | Gravel | 1.000 | 0.135 | 0.252 |
| Sand | | 0.205 | 0.751 | | Sand | | 0.637 | <0.001 | | Sand | | <0.001 | 0.223 | | Sand | | 0.230 | 0.414 |
| Silt | | | 0.015 | | Silt | | | <0.001 | | Silt | | | 0.001 | | Silt | | | 0.916 |
| | | | | | | | | | | | | | | | | | | |
| Summer | Sand | Silt | Vegetation | | Summer | Sand | Silt | Vegetation | | Summer | Sand | Silt | Vegetation | | Summer | Sand | Silt | Vegetation |
| Gravel | 0.355 | 0.001 | 0.530 | | Gravel | 0.001 | 0.984 | 0.951 | | Gravel | 0.002 | 0.087 | 0.945 | | Gravel | 0.038 | 0.442 | 0.297 |
| Sand | | 0.131 | 0.992 | | Sand | | 0.001 | 0.004 | | Sand | | 0.574 | 0.011 | | Sand | | 0.638 | 0.001 |
| Silt | | | 0.066 | | Silt | | | 0.810 | | Silt | | | 0.277 | | Silt | | | 0.007 |
| | | | | | | | | | | | | | | | | | | |
| Autumn | Sand | Silt | Vegetation | | Autumn | Sand | Silt | Vegetation | | Autumn | Sand | Silt | Vegetation | | Autumn | Sand | Silt | Vegetation |
| Gravel | 0.154 | 0.794 | 0.004 | | Gravel | <0.001 | 0.896 | 0.967 | | Gravel | 0.026 | 0.915 | 0.775 | | Gravel | 0.977 | 0.015 | 0.795 |
| Sand | | 0.014 | <0.001 | | Sand | | <0.001 | <0.001 | | Sand | | 0.003 | 0.001 | | Sand | | 0.004 | 0.548 |
| Silt | | | 0.063 | | Silt | | | 0.996 | | Silt | | | 0.990 | | Silt | | | 0.166 |

## Slide 6
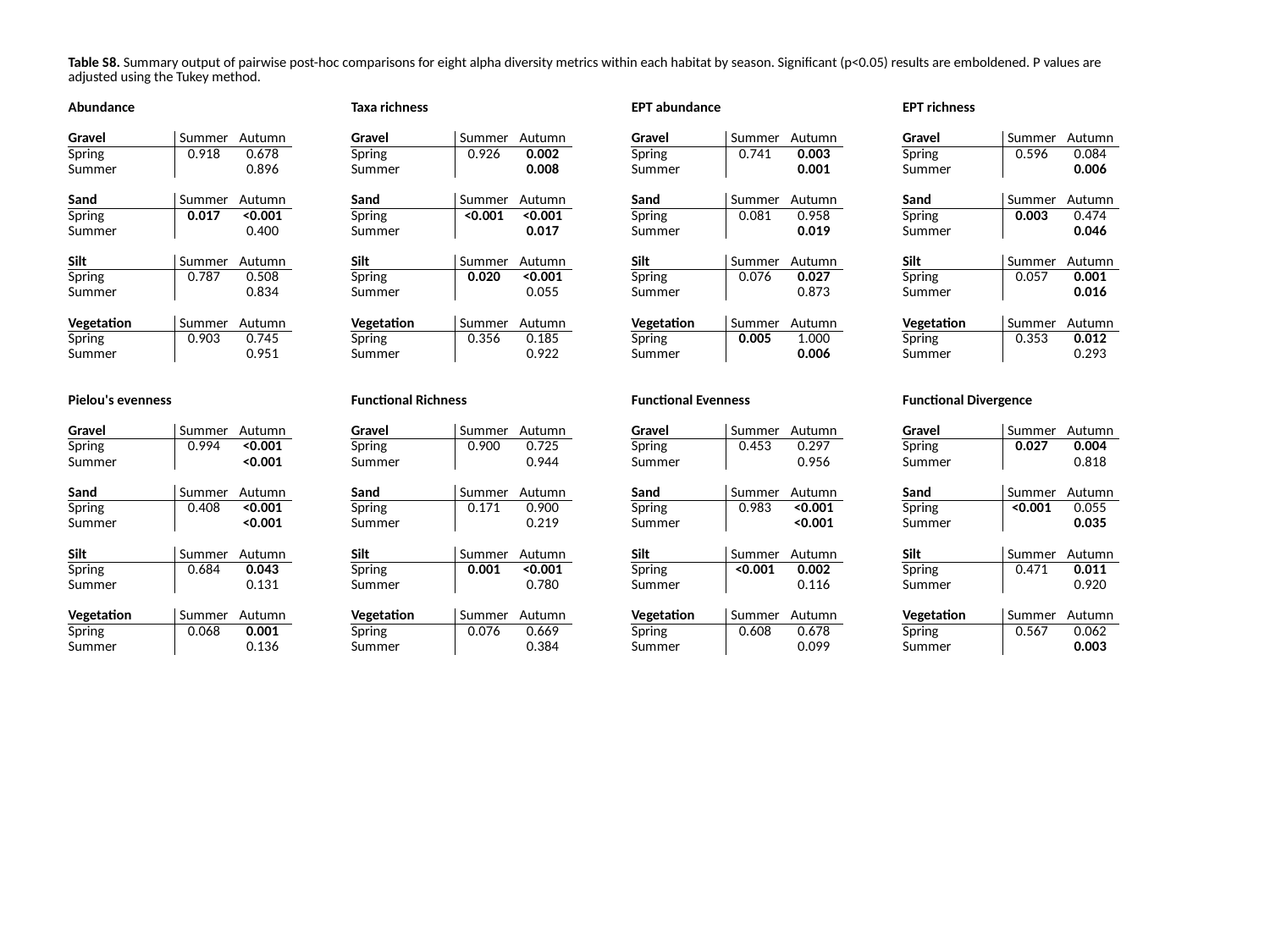

| Table S8. Summary output of pairwise post-hoc comparisons for eight alpha diversity metrics within each habitat by season. Significant (p<0.05) results are emboldened. P values are adjusted using the Tukey method. | | | | | | | | | | | | | | |
| --- | --- | --- | --- | --- | --- | --- | --- | --- | --- | --- | --- | --- | --- | --- |
| | | | | | | | | | | | | | | |
| Abundance | | | | Taxa richness | | | | EPT abundance | | | | EPT richness | | |
| | | | | | | | | | | | | | | |
| Gravel | Summer | Autumn | | Gravel | Summer | Autumn | | Gravel | Summer | Autumn | | Gravel | Summer | Autumn |
| Spring | 0.918 | 0.678 | | Spring | 0.926 | 0.002 | | Spring | 0.741 | 0.003 | | Spring | 0.596 | 0.084 |
| Summer | | 0.896 | | Summer | | 0.008 | | Summer | | 0.001 | | Summer | | 0.006 |
| | | | | | | | | | | | | | | |
| Sand | Summer | Autumn | | Sand | Summer | Autumn | | Sand | Summer | Autumn | | Sand | Summer | Autumn |
| Spring | 0.017 | <0.001 | | Spring | <0.001 | <0.001 | | Spring | 0.081 | 0.958 | | Spring | 0.003 | 0.474 |
| Summer | | 0.400 | | Summer | | 0.017 | | Summer | | 0.019 | | Summer | | 0.046 |
| | | | | | | | | | | | | | | |
| Silt | Summer | Autumn | | Silt | Summer | Autumn | | Silt | Summer | Autumn | | Silt | Summer | Autumn |
| Spring | 0.787 | 0.508 | | Spring | 0.020 | <0.001 | | Spring | 0.076 | 0.027 | | Spring | 0.057 | 0.001 |
| Summer | | 0.834 | | Summer | | 0.055 | | Summer | | 0.873 | | Summer | | 0.016 |
| | | | | | | | | | | | | | | |
| Vegetation | Summer | Autumn | | Vegetation | Summer | Autumn | | Vegetation | Summer | Autumn | | Vegetation | Summer | Autumn |
| Spring | 0.903 | 0.745 | | Spring | 0.356 | 0.185 | | Spring | 0.005 | 1.000 | | Spring | 0.353 | 0.012 |
| Summer | | 0.951 | | Summer | | 0.922 | | Summer | | 0.006 | | Summer | | 0.293 |
| | | | | | | | | | | | | | | |
| | | | | | | | | | | | | | | |
| Pielou's evenness | | | | Functional Richness | | | | Functional Evenness | | | | Functional Divergence | | |
| | | | | | | | | | | | | | | |
| Gravel | Summer | Autumn | | Gravel | Summer | Autumn | | Gravel | Summer | Autumn | | Gravel | Summer | Autumn |
| Spring | 0.994 | <0.001 | | Spring | 0.900 | 0.725 | | Spring | 0.453 | 0.297 | | Spring | 0.027 | 0.004 |
| Summer | | <0.001 | | Summer | | 0.944 | | Summer | | 0.956 | | Summer | | 0.818 |
| | | | | | | | | | | | | | | |
| Sand | Summer | Autumn | | Sand | Summer | Autumn | | Sand | Summer | Autumn | | Sand | Summer | Autumn |
| Spring | 0.408 | <0.001 | | Spring | 0.171 | 0.900 | | Spring | 0.983 | <0.001 | | Spring | <0.001 | 0.055 |
| Summer | | <0.001 | | Summer | | 0.219 | | Summer | | <0.001 | | Summer | | 0.035 |
| | | | | | | | | | | | | | | |
| Silt | Summer | Autumn | | Silt | Summer | Autumn | | Silt | Summer | Autumn | | Silt | Summer | Autumn |
| Spring | 0.684 | 0.043 | | Spring | 0.001 | <0.001 | | Spring | <0.001 | 0.002 | | Spring | 0.471 | 0.011 |
| Summer | | 0.131 | | Summer | | 0.780 | | Summer | | 0.116 | | Summer | | 0.920 |
| | | | | | | | | | | | | | | |
| Vegetation | Summer | Autumn | | Vegetation | Summer | Autumn | | Vegetation | Summer | Autumn | | Vegetation | Summer | Autumn |
| Spring | 0.068 | 0.001 | | Spring | 0.076 | 0.669 | | Spring | 0.608 | 0.678 | | Spring | 0.567 | 0.062 |
| Summer | | 0.136 | | Summer | | 0.384 | | Summer | | 0.099 | | Summer | | 0.003 |
